# Computer-Aided Drug Design (CADD) to De-Orphanize Marine Molecules: Finding Potential Therapeutic Agents for Neurodegenerative and Cardiovascular Diseases

**DOI:** 10.3390/md20010053

**Published:** 2022-01-05

**Authors:** Laura Llorach-Pares, Alfons Nonell-Canals, Conxita Avila, Melchor Sanchez-Martinez

**Affiliations:** 1 Mind the Byte S.L., 08028 Barcelona, Catalonia, Spain; lllorak@gmail.com (L.L.-P.); alfons.nonell@devshealth.com (A.N.-C.); 2 Department of Evolutionary Biology, Ecology and Environmental Sciences, Faculty of Biology and Biodiversity Research Institute (IRBio), University of Barcelona, 08028 Barcelona, Catalonia, Spain; conxita.avila@ub.edu

**Keywords:** marine natural products, computer-aided drug design, virtual profiling, neurodegenerative diseases, cardiovascular diseases

## Abstract

Computer-aided drug design (CADD) techniques allow the identification of compounds capable of modulating protein functions in pathogenesis-related pathways, which is a promising line on drug discovery. Marine natural products (MNPs) are considered a rich source of bioactive compounds, as the oceans are home to much of the planet’s biodiversity. Biodiversity is directly related to chemodiversity, which can inspire new drug discoveries. Therefore, natural products (NPs) in general, and MNPs in particular, have been used for decades as a source of inspiration for the design of new drugs. However, NPs present both opportunities and challenges. These difficulties can be technical, such as the need to dive or trawl to collect the organisms possessing the compounds, or biological, due to their particular marine habitats and the fact that they can be uncultivable in the laboratory. For all these difficulties, the contributions of CADD can play a very relevant role in simplifying their study, since, for example, no biological sample is needed to carry out an in-silico analysis. Therefore, the amount of natural product that needs to be used in the entire preclinical and clinical study is significantly reduced. Here, we exemplify how this combination between CADD and MNPs can help unlock their therapeutic potential. In this study, using a set of marine invertebrate molecules, we elucidate their possible molecular targets and associated therapeutic potential, establishing a pipeline that can be replicated in future studies.

## 1. Introduction

**Figure 1:**
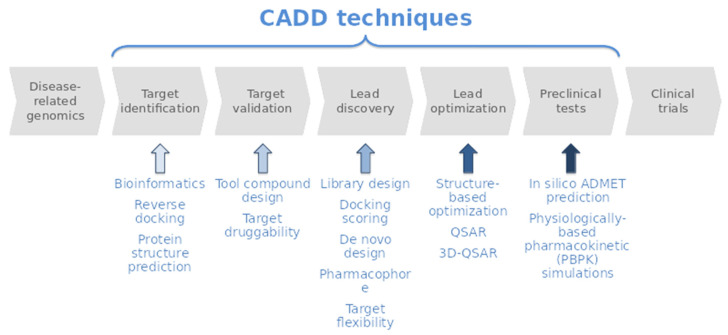
Schematic pipeline of the Drug Discovery cycle highlighting where CADD techniques are used.

In the context of drug discovery, the progression from basic research to market through the various pre-clinical and clinical phases takes tens of years and costs billions of dollars [1]. This constitutes a huge problem for the development of new treatments, ultimately becoming a social problem because of its potential influence on reducing the high number of unmet medical needs. However, the usage of Computer-aided drug discovery/design (CADD) techniques, used at different stages of the drug discovery process (Figure 1), helps to mitigate this overall cost [2]. Hence, CADD methods are becoming very popular tools and have played an important role over the last decades in the development of therapeutically important molecules. [2,3,4,5]. They are mostly used in the pre-clinical phase, although there is increasing potential for their use in clinical phases [6].

CADD techniques cover various aspects of the drug discovery process, from the selection of candidate molecules to the optimization of lead compounds [4,7] (Figure 1). They are usually classified into Ligand-Based (LB) and Structure-Based (SB) techniques depending on the nature of the input molecule [4,7]. Structure-based methods use the 3D structure of the target (protein) as a key element for the generation or screening of potential ligands (modulators), which can be then converted into hit compounds (molecules with biological activity). The ligand-based approach considers a set of molecules with diverse structures and known potency, used to find hit compounds by developing theoretical prediction models to, for example, find chemical analogues or biomimetic compounds to a known substrate, inhibitor, or drug. The potency, selectivity, and ADMET properties of the selected compounds are then optimized (in silico and even in vitro in some cases) to convert them into leads, which are then experimentally validated (in vitro and in vivo) to become drug candidates. Both types of methods, although distinct, are often used together. The combination of different structure- and ligand-based design strategies is more effective than any single approach, as both methods complement each other’s strengths and weaknesses [2,4,7].

An example of this combination of methodologies is, for example, the use of molecular modelling techniques such as docking and molecular dynamics (MD) to validate virtual profiling (VP) experiments, as shown in this work. VP methods can predict the biological profile and mechanisms of action (MoA) of a given molecule, indicating its most likely targets. Target deconvolution is a crucial early step in drug development to determine the focus and strategy of the research. In this regard it is frequently used to predict the possible targets of an orphan ligand or the most similar targets to an investigational protein [8]. Docking and MD methods can predict ligand–target interactions in terms of binding mode and/or binding strength, also allowing discrimination between target–ligand molecular associations. Molecular docking and MD methods are very popular tools used to evaluate ligand–target complementarity [5,9,10,11,12]. These methods allow the study of protein–ligand interactions at an atomic level as well as the prediction of preferred binding orientation or poses (binding mode) of one molecule (typically a small organic compound) to another molecule (generally a biological target such as a protein). They also allow to estimate the binding affinity of the ligand to the target [11,13,14]. The combination allows the results of VP experiments to be more robust, enabling the reduction of false positive results.

CADD techniques have been applied to a wide variety of molecules and diseases, including natural products (NPs). NPs play a key role in drug discovery and their identification is one of the most promising lines of drug discovery [15,16,17,18]. Over the years, there are many examples of drugs that have emerged from natural products (secondary metabolites) and derivatives of these products [18]. NPs present an enormous chemical diversity with vast therapeutic potential. Among natural products, those from the sea are particularly interesting in drug discovery because of their novelty due to the highly unexplored marine world. This lack of exploration, coupled with the fact that the oceans are home to most of the world’s biodiversity, means that marine natural products (MNPs) may have novel and potentially very useful chemical structures [19,20].

Oceans cover about 70% of the Earth’s surface and up to 80% of life inhabits them, offering a huge diversity of enzymes and bioactive compounds that can be exploited in different areas, including drug discovery [20,21]. Biodiversity directly relates to chemodiversity, providing huge opportunities for discovering novel therapeutics with novel mechanisms of action [15,16,20,21]. Marine natural products (MNPs) have been a rich source of drug-like compounds for decades [16]. In 2020, there were eight marine-derived products approved as drugs by the United States Food and Drug Administration (US FDA) or the European Medicines Agency (EMA) [22]. In addition to that, tens of drug candidates are in phase I-III of drug development, highlighting the increasing potential of MNPs [16,22,23]. Once the compounds have been obtained, their complex structures and their molecular targets can be difficult to establish, particularly when the amount of compounds is very small [15,24,25,26,27]. All these factors must be added to the proper implementation of the Nagoya Protocol and the bioavailability of marine natural products, making computational techniques particularly useful in these cases. In silico CADD techniques help to reduce the number of experiments needed and, in addition, do not require biological samples, helping to overcome, at least partially, the above-mentioned difficulties and to protect biodiversity in sensitive habitats.

**Figure 2:**
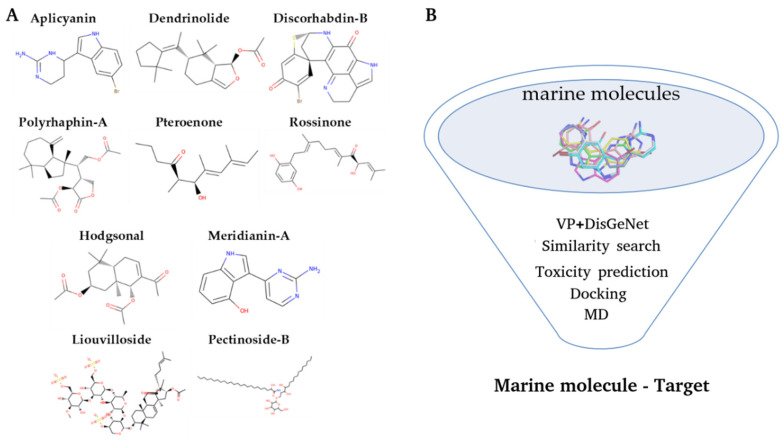
(**A**) Structures of the ten marine molecules selected for this study (**B**) Graphical representation of the workflow process followed to the exploration of all set of marine molecules.

In this study, a chemical library of marine molecules from benthic invertebrates was explored, setting up an initial set of 10 molecules, as explained in the methods section. (Figure 2). Based on the idea of that marine molecules are a rich source of potential drugs [17,18,21,28] and on the fact of CADD techniques have been used to successfully de-orphanize NPs (from terrestrial and marine origin) [29,30,31,32,33,34], the aim of the present study is **(1)** to disentangle the possible therapeutic potential of a set of marine molecules, as well as **(2)** to devise a plausible computational workflow for future studies. To do that, three main milestones were established: (1) to elucidate a list of possible targets, performing two dimensions (2D) and three dimensions (3D) ligand-based virtual profiling (VP) experiments; (2) to evaluate the possible toxicity and drug-likeness of the compounds by using QSAR models as well as a comparing their behavior with known drugs; (3) to determine the binding mode and binding energy of the protein–compound complexes by using classical and blind docking, as well as MD simulations. This study has been conducted blindly, without any prior information on possible therapeutic applications, to better exemplify the potential of computational approaches.

## 2. Results and Discussion

### 2.1. Virtual Profiling

Using Cabrakan and Hurakan software tools [35,36], 2D and 3D virtual profiling studies (based on molecular similarity) over 8 of the 10 molecules set were performed. Two molecules of the initial set, Liouvilloside and Pectinoside-B, were discarded because they were too big to be analyzed by Hurakan (molecules with a molecular weight > 900 Da and rotatable bonds > 32 are discarded). Before their complete withdrawal we analyzed them with Cabrakan (less restrictive than Hurakan regarding molecule size), but no targets were obtained for them. For the remaining eight molecules, different targets (summarized in Table 1) were predicted, except for Discorhabdin B for which no targets were found. These kinds of studies rely on the database over which the similarity searches are performed. Some marine molecules used to have complex structures from a chemical point of view, therefore it is not strange that similar molecules are not found in a general chemical database, since they are not specifically based on marine entities. As VP experiments typically predict many targets, we only selected the most relevant, those that have at least three matches in the databases, giving relevance to the similarity and ensuring the viability of the complex.

**Figure 3:**
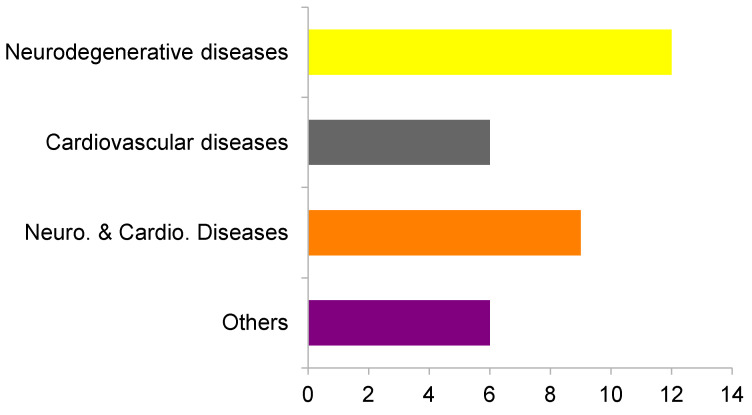
Relation between selected targets and analyzed pathologies. Yellow: neurodegenerative, Grey: cardiovascular, Orange; Neurodegenerative and cardiovascular, Purple: Other pathologies.

Thereafter, by using DisGeNET [37], we checked the possible therapeutic indication of each molecule by analyzing the diseases on which the predicted targets may be involved. A total of 12 targets were related to neurodegenerative diseases and six to cardiovascular diseases. Additionally, nine targets were related to both neurodegenerative and cardiovascular diseases. Six targets were also related to other disorders, specifically orphan diseases (digital clubbing, pituitary-dependent Cushing’s disease, and mental retardation X-linked), peripheral nervous system diseases, as well as prostatic and lung neoplasms (Figure 3). After analyzing the results, due to their prevalence among them and their high social impact as well as our own research interests, we decided to focus on neurodegenerative and cardiovascular diseases. 

According to the World Health Organization (WHO) there are around 50 million people that have dementia and nearly 10 million new cases every year [38]. Since humans’ life expectancy continues to increase, consequently, the number of patients increases every year. This therapeutic area has been commonly described as one of the more pressing and frustrating. The difficulty of its study is marked by several factors, including its heterogeneity, the fact that there is still much to learn about its pathogenesis and the difficulties associated with its diagnosis, which is usually quite late, limiting the power of intervention. It is therefore imperative to pursue any drug discovery approach that appears promising in terms of supporting hypotheses and clear targets [39,40]. In this context, the potential of NPs in neurodegenerative diseases has been confirmed by several studies over the years, including some recent ones using MNPs [26,40,41]. Hence, it constitutes a great potential opportunity for the molecules studied here.

Cardiovascular diseases are considered the first cause of death globally, with a resurgence in high-income countries, and a rapid and steady increase in low- and middle-income countries, taking an estimated 18 million lives each year [42,43]. Initiatives are needed to accelerate the discovery and development of new therapies. It is generally accepted that there is a gap in unravelling new mechanisms that may be particularly relevant to differences in individual susceptibility and resistance to common cardiovascular diseases. There is great potential to fill these gaps by applying proven drug discovery methods to novel and mechanistically robust targets [43]. In this regard, marine drugs have emerged as potential therapeutic opportunities [26,44,45]. All this makes cardiovascular diseases interesting potential targets for the molecules studied here.

After VP experiments, 32 targets, corresponding to seven molecules, were selected. Some of these targets were predicted for more than one molecule within the set. Specifically, there were nine targets that could interact with more than one marine molecule. Therefore, in the end, we established 75 possible marine molecule–target complexes.

### 2.2. Toxicity Prediction

Many drugs fail in the clinical phases because of toxicity problems. Toxicity is, at best, firstly evaluated after the hit, or even the lead, identification phase. Therefore, some candidates obtained after a non-negligible number of experiments have to be discarded. Conducting a toxicity analysis in the early stage of the drug discovery process could help to avoid large investments of time and money in a drug that will be unfeasible. At the same time, it allows scientists to better focus their experiments on the most promising compounds from the beginning.

Accordingly, we analyzed the potential toxicity issues of the 10 molecules from the outset. Carcinogenicity, mutagenicity, developmental toxicity, and propensity to skin sensitization was assessed using VEGA QSAR models [46]. Of the set of molecules, Liouvilloside was outside the applicability domain of the models, and consequently no results were obtained. For the rest of the molecules, predictions were made (with varying degrees of confidence) (Table 2).

In addition, we performed an additional check based on molecular similarity to known toxins. A Tanimoto-based 2D similarity analysis of the set of molecules was performed on the toxin and toxin target database (T3DB) (Figure S1). However, none of the molecules had significant similarity to any toxin in the database. Therefore, no toxicity can be extrapolated from these analyses, as our marine molecules are not similar to the known toxins in the database.

Our results indicate that all the marine molecules analyzed here appear to have a certain toxic propensity, albeit low in most cases. These results can be explained by the fact that these marine molecules originate from marine organisms that use them as chemical defenses against potential predators and competitors [47]. A similar example is the well-known Paclitaxel, Taxol by trade name, a chemical defense alkaloid with toxic effects on mammalian cells that has become a highly used anticancer chemotherapy drug [48]. 

In terms of skin sensitisation, Hodgsonal and Pteroenone may be particularly problematic, as well as Rossinone-A to a lesser extent. In addition, Hodgsonal and Polyrhaphin-A may cause developmental toxicity problems and, together with Pteroenone, present carcinogenicity issues. With these results in hand, Aplicyanin, Meridianin-A, Dendrinolide, Discorhabdin-B, and Pectinoside-B showed the safest toxicity profiles, while Hodgsonal, Polyrhaphin-A, Pteroenone, and to a lesser extent Rossinone-A, present more worrying profiles. Despite this, we decided not to discard any compound after these experiments, as the observed toxicity profiles are quite similar between all compounds, and there was a manageable amount of protein–ligand complexes.

### 2.3. Virtual Profiling Validation. In Silico Binding Studies

#### 2.3.1. Docking and Molecular Dynamics Simulations

After analyzing the potential toxicity of the compounds, docking calculations were performed to (1) check how they bind (docking) with respect to known drugs of the predicted targets (Table S7) and to (2) validate VP results. For this, the binding of the 75 predicted target–molecule complexes were analyzed in terms of binding energy and binding mode. From the docking results, the most promising complexes (those with a binding energy lower than −6.5 Kcal/mol) were selected for further studies. Fifty-two complexes (corresponding to 30 targets) were selected and 23 were discarded. Of the 52 complexes, 36 were studied from crystallographic structures and the remaining 16 from homology models (Tables S2–S4). 

**Figure 4:**
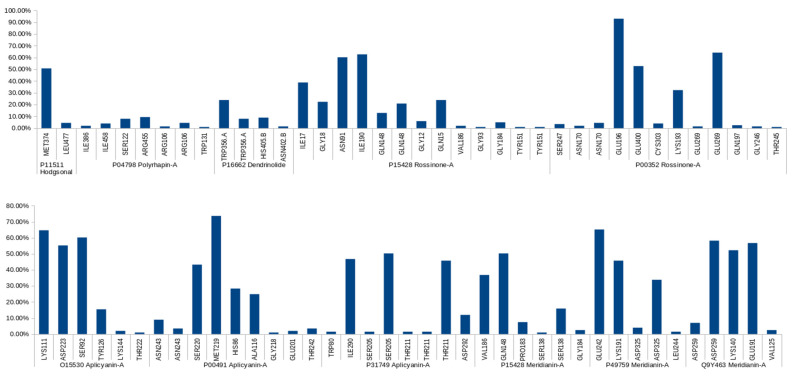
The graph shows the hydrogen bond (HB) occupancy per target. Only the best molecule–target complexes (Table S5) are reported. Residue numbers correspond to Wild Type sequence numbering from Uniprot. All those occupancies lower than 0.99% were not taken into account and are not shown. Horizontal numbers are the Uniprot ID, and vertical letters and numbers refer to the residue involved on the HB of each target. If a residue appears several times it means that different HBs have been detected between the ligand and the residue.

The 52 best complexes were subjected to short MD simulations to post-process the docking poses in order to add protein flexibility to the equation. When analyzing the MD results to select the best complexes (in terms of complex stability as well as observed binding mode and binding energy), pteroenone was discarded because an artefact was detected during the analysis of the simulations. For the rest of the molecules, different complexes were selected. In some cases, more than one complex per molecule. For Hodgsonal, Polyrhaphin-A and Dendrinolide, a complex targeting P11511, P04798, and P16662, respectively, was selected. Two complexes targeting P15428 and P00352 were selected for Rossinone-A. For Meridianin-A, three complexes targeting Q9Y463, P15428, and P49759 were selected, and for Aplicyanin-A three complexes with the targets O15530, P00491, and P31749 were selected. In summary, 11 marine molecule–target complexes were selected, 10 targets (one of them twice) with six molecules (Table S5). All simulations performed were stable; the simulated complexes were maintained during the generated trajectories, and no ligand dissociated from the target (Figures S2–S6). The stability of the complexes was also refuted by the analysis of the interactions because it revealed the presence of strong polar interactions and hydrogen bonds (Figure 4, Table S6).

#### 2.3.2. Binding Mode Analysis. Hydrogen Bonding

Hydrogen Bonds (HBs) have been proposed to act as structural anchors of protein–ligand complexes [49,50]. Their presence has been related with complex stability and their analysis is a good way to approximate the stability of marine molecule–target complexes. In this regard, the time-averaged number of HBs present on each marine molecule–target complex was calculated from the MD trajectories. HBs were defined in such a way that the distance between donor and acceptor was less that the cut-off distance of 3.5 Armstrongs (0.35 nm) and the angle donor-H-acceptor was less than the cut-off angle of 20 degrees.

In addition to assessing the possible stability of the marine molecule–target complex, the analysis of the HBs allows us to compare the binding modes obtained with the reported interactions for known substrates or inhibitors of the targets studied. In this way, the viability of the predicted complexes is also evaluated.

Looking at the resulting HB occupancy during the MD simulation of each complex, it can be observed that for most of the studied target–ligand complexes we found stable interactions along the trajectory (Figure 4, Table 3). This is consistent with the fact that no ligand dissociated from the target during MD.

Protein–ligand complexes are dynamical systems, and MDs reflect this behavior. Thus, HBs during MD are not always constant. They can break and form constantly. The HBs found were classified according to their live time as long-lived (present in more than 50% of the simulation), medium-lived (present between 10% and 50%), and short-lived (formed in less than 10% of the simulation) [51,52]. 

Most of the reported long-lived HBs have also been identified as key residues for the binding of known ligands to the targets predicted for marine compounds. **Hodgsonal-P11511** HB with MET374 is also established by the natural substrate and other inhibitors [53,54,55,56]. **Aplicyanin-A-O15530** HBs with ASP223 and LYS111 are also established by known inhibitors [57,58]. Analyzing adenosin triphoshate (ATP), O15530 natural substrate, containing PDB structures a HB with SER92 is also observed [59,60,61] as for Apliacynin-A. **Aplicyanin-A-P31749** HB with SER205 has also been observed in other compounds binding to the same binding site [62,63]. **Aplicyanin-A-P00491** HB with MET219 has been reported as a key interaction for other compounds [64,65,66]. HBs with SER220 has also been observed in the binding site [66,67,68,69]. **Rossinone-A-P00352** HBs with GLU196, GLU269 and GLU400 are also established by the natural cofactor within the orthosteric site [70]. For **Rossinone-A-P15428**, a HB with ASN91 is also established by other reported compounds [71,72,73,74]. **Meridianin-A-P15428** HB with GLN148 is also established by the natural substrate [71,72,73,74]. For **Merdianin-A-Q9Y463**, HBs with LYS140 and GLU191 are also reported for other inhibitors in a homologous protein Q13627 (DYRK1A) numbered as LYS188 and GLU239 [75,76,77]. Q13627 and Q9Y463 (DYRK1B) vary by only one amino acid in the ATP-binding pocket [78,79]. Due to this high similarity, there are compounds that have also been reported as dual Q13627 and Q9Y463 inhibitors [76,77,78]. **Meridianin-A-P49759** HB with GLU242 have been previously reported for other compounds binding to P49759 [80,81,82]. To sum up, 87.5% of the predicted long-lived HBs (14 of 16) were reported in the literature. This fact strongly supports our target predictions and validates our approach.

In addition to the reported long-lived HBs, other short- and medium-lived HBs have also been identified as important for the binding of known compounds to their respective targets (Table 3). As commented before MDs reflect the dynamical nature of protein–ligand associations. Occasionally, in the literature, the reported binding modes come from a static representation from one snapshot of an NMR study or from an X-ray crystal. Thus, this reported binding mode can incorporate, as can our MDs, interactions with a different life-length. In this regard, the fact that short and medium-lived HBs have also been reported should be considered as an additional support to our results and methodology. Furthermore, the detection of key HBs (Table 3), regardless of their lifetime, can be considered as a signal that we are acting at the right binding site. 

Unfortunately, there are complexes for which long-term HBs were not predicted. Complexes in which no long-lived HB was found should be treated with caution. This may be a consequence of the system requiring longer equilibration, or an indication that the binding mode is unreliable or difficult to observe naturally. For **Dendrinolide-P16662**, no long-lived HB was found. In this case, the reason could be that the binding mode where the molecules were predicted to bind is not correct. 2O6L [83], the 3D structure used, is a dimer and the reported interactions/binding mode correspond to the interaction region of the monomers. TRP356 (with which a medium-lived HB has been detected) is a residue that according to other members of the protein family forms the putative substrate, UDPGA, interacting binding site. However, Dendrinolide is located at the border of this binding site but outside of it. In the case of the **Polyrhaphin-A-P04798**, no long-lived HB was found either. The residues that perform short-lived interactions are within the binding site. However, it appears that some of these residues are located in the cofactor binding region, rather than the inhibitor binding region [84,85,86,87]. In this case, the binding mode could be correct, but the biological effect of targeting the cofactor binding region should be further analyzed, as we were originally expecting to compete against substrates and not cofactors. There are examples where competing with the cofactor is a valid strategy [88,89], but for this target we believe it would not be a good strategy and in fact to our knowledge there are no examples in the literature.

It can be seen that P15428 has been studied twice, with Meridianin-A and Rossinone-A. According to the results, the residues involved in the formation of HBs in the two complexes are not the same, except for GLN148. This is probably due to the size of the binding cavity, which is quite large, favoring the existence of different binding patterns within it. In addition, the different flexible nature of the molecules, as Rossinone is much more linear, may also be a reason for these differences. It appears that Rossinone-A binding may overlap with the NAD+ cofactor, as it binds within the catalytic site but between the cofactor and substrate binding regions, interacting with residues from both regions as they are next to each other [70,71]. Meridianin-A binds to the substrate/inhibitor binding region and, in principle, this binding mode is much more reasonable. Reported inhibitors with activities in the low nM range have shown a binding mode similar to that of Meridianin-A [72]. In addition, the presence of NAD+ seems to be necessary to alter the local electrostatic environment, which favors inhibitor binding [92]. Further analysis, including simulations with the cofactor present, would be interesting and necessary to fully understand whether Rossinone-A could bind to the “right site” and behave as an inhibitor or whether its predicted binding is just an artefact due to the lack of the cofactor in the simulation. However, this is beyond the scope of this study.

Rossinone-A is also involved in the binding to another cofactor region; namely with P00352, the ALDH1 protein. However, the inhibitors developed bind to the substrate binding site [93,94,95,96]. In this case, binding to the cofactor region may be of some interest. The cofactor binding domain, NAD, shares a motif (Rossman-fold) with other dehydrogenases. There are differences in the motif between ALDH and other families of NAD+-binding enzymes. It has been proposed that this situation could be exploited for the development of small-molecule modulators of various ALDH isozymes compared to other NAD+-dependent dehydrogenases [70,97]. However, this binding site is highly conserved within the ALDH family and the development of selective modulators targeting it can be difficult. Some studies even avoid identifying compounds that interact at the cofactor binding site as, for example, compounds binding are less likely to be selective for members of the ALDH1/2 class [70]. Therefore, it is not too interesting to further explore this binding mode.

In any case, it is interesting to note that some compounds are predicted to apparently bind to the cofactor binding region. As discussed above, this could be a valid strategy, depending on the target and the biological effect sought, but compounds are usually more likely to bind to the substrate region. In general, and in particular for the targets studied here, complexes showing such cofactor interactions are usually less interesting and should be treated with caution. Furthermore, this indicates a possible gap in our workflow. There seems to be a tendency to predict wrong binding modes in cases where cofactors are involved in the catalytic activity of the target. Including the cofactor in the simulations would probably alleviate this problem.

#### 2.3.3. Binding Mode Analysis. Molecular Mechanics/Generalized Born Surface Area (MM/GBSA). Overall Molecule–Target Association

**Figure 5:**
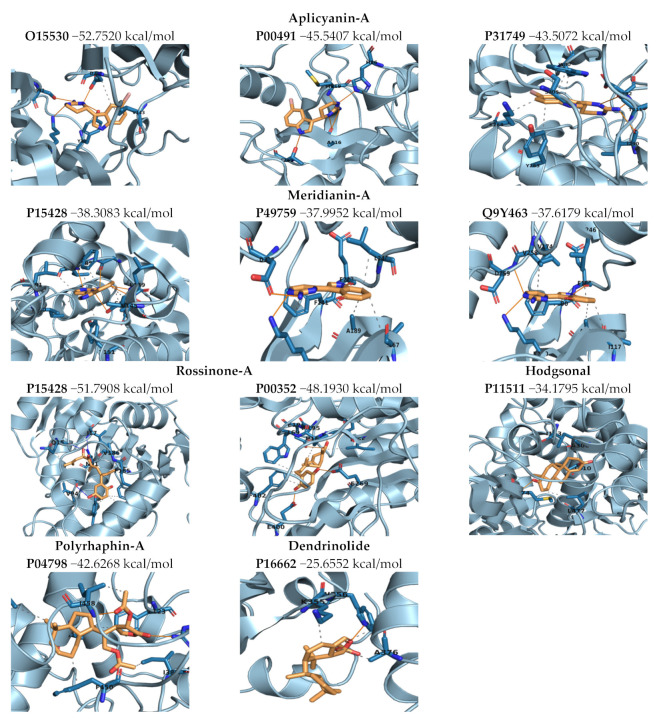
Images of the binding mode of each marine molecule inside the binding cavity of the corresponding target (last frame of the trajectory). Marine molecules and interacting residues are represented in sticks, while proteins are shown as cartoons. Orange lines indicate HBs, grey dashed lines hydrophobic interactions. Binding energies obtained by MM/GBSA calculations are reported next to the target name.

Finally, to more accurately quantify the ligand–protein binding energy, Molecular Mechanics/Generalized Born Surface Area (MM/GBSA) calculations over the generated MD trajectories for post-processing docking results were performed (Figure 5). These kinds of simulations allow us to estimate more realistic binding energies than rigid docking calculations, as protein and ligand flexibility are taken into account [12,98,99,100]. However, although more realistic, the results are not directly comparable to experimental results. 

Looking at post-processed binding energies (Figure 5), results indicate that all the complexes showed good values and thus the molecule–target interaction could be plausible from an energetic point of view (in line with previous structural analysis/validations). This energetically favorable scenario is also confirmed from a structural point of view. As mentioned above, interactions were predicted for the Aplicyanine and Meridianine complexes, as well as for Hodgsonal, which are in agreement with the literature. However, in other cases, although good energy was obtained, the predicted binding mode is not correct or, at least, rather difficult to observe in nature and/or to exploit for therapeutic purposes.

Aplicyanin-A was predicted to interact with three targets: O15530, P00491, and P31749. The binding strength to the three targets is quite similar, but a slight preference is shown for O15530. P31749 and O15530 are two Serine/Threonine kinase, 3-phosphoinositide-dependent protein kinase 1 (PDK1), and RAC-alpha serine/threonine–protein kinase (AKT1), respectively. P00491, Purine Nucleoside Phosphorylase (PNP), is an enzyme involved in purine metabolism. 

AKT1 is involved in several diseases including CNS disorders, like schizophrenia or Alzheimer, and cancer, like breast cancer and prostate cancer [101,102]. Many cellular processes, such as cell survival, are affected by Akt. Its inhibition is pursued in cancer but its presence and function are sought in CNS diseases. In this study, we observed that Aplicyanin-A behaves like well-known allosteric inhibitors. Thus, Aplicyanin-A related to AKT1 is more likely an anti-cancer agent rather than a therapeutic option for CNS disorders. In any case, it would be interesting to analyze the binding of Aplicyanin-A to the orthosteric site of AKT1 to check if this could change the commented behavior.

PDK1 deficiency causes cardiac problems [103,104,105,106]. However, its inhibition also has beneficial effects, for example in the treatment of cancer [107,108]. Both activators and, above all, inhibitors of PDK1 have been described in the literature [107,109]. Activators have been shown to bind to the small lobe of the kinase domain (so-called HM/PIF binding pocket), exerting their activity allosterically [109]. However, Aplicyanin-A has been predicted to bind to the orthosteric ATP binding site, establishing interactions similar to known inhibitors. Therefore, PDK1-related Aplicyanin-A appears to be an anticancer agent rather than a therapeutic option for cardiovascular diseases. In any case, it would be interesting to analyze the binding of Aplicyanin-A to the allosteric site of PDK1 to check if Aplicyanin-A could act as an activator.

PNP is involved in nucleoside metabolism. Nucleoside receptors are known to be important targets for a variety of brain diseases [110]. For example, the G51S polymorphism of PNP has been linked to cognitive impairment in Alzheimer’s disease (AD). However, PNP is also implicated in other diseases and its inhibitors have been nominated as a possible approach for the treatment of cancers and autoimmune diseases, including gout, rheumatoid arthritis, psoriasis, tissue transplant rejection, and multiple sclerosis [65,111]. Among the different diseases with which PNP is associated, cancer treatment is the main reported use for PNP inhibitors. Apliacanin-A has been predicted to bind to the orthosteric site, establishing interactions similar to those of reported anticancer inhibitors. Therefore, Apliacynin-A related to PNP appears to be more likely to exhibit anti-cancer activity than to act against neurodegenerative diseases. In any case, it would be interesting to analyze the binding of Aplicyanin-A to the allosteric site (reported in the literature [112]) to check whether binding is possible and whether it could affect the observed behavior.

The anticancer nature of Aplicyanins has been reported in the literature [113,114,115]. Therefore, it makes sense that they act as anticancer agents rather than as compounds with anti-neurodegenerative or cardiovascular disease-related effects.

Meridianin-A was predicted to interact with three targets: P15428, P49759, and Q9Y463. Simulations show no significant difference in binding energy, suggesting that Meridianin-A may not be selective for these targets and could lead to potential selectivity problems. P49759, Dual specificity, cdc2-like, protein kinase 1 (CLK1), and Q9Y463, Dual specificity tyrosine-phosphorylation-regulated kinase 1B (DYRK1B) are kinases acting on both serine/threonine and tyrosine-containing substrates. Both belong to the so-called dual specificity kinases (EC 2.7.12.1) which include CLKs (1–4) and DYRKs (1A,1B, 2–4), among others. They are related to different diseases, including neurodegenerative disorders, cancer, or diabetes [116]. Meridianin-A binds to the orthosteric, ATP, binding site in both cases, establishing similar interactions as reported inhibitors. P15428, 15-hydroxyprostaglandin dehydrogenase [NAD(+)] (HPGD), is an enzyme involved in the regulation of events that are under the control of prostaglandin levels. In this case, Meridianin-A is predicted to bind to the substrate/inhibitor region of P15428, not to the cofactor binding place as Rossinone-A does, in agreement with known inhibitors.

As commented above, CLK1 is involved in several diseases, including neurodegenerative disorders. It has been designed as a novel target for AD [81]. In this regard it is involved, together with other kinases, in tau phosphorylation. Inhibition of specific tau kinases or kinases involved in tau phosphorylation pathway is considered a promising strategy to fight against AD [117]. The main protein kinases involved in tau phosphorylation have been grouped into two classes: tau protein kinases, GSK3β and CK1δ, and dual specificity kinases, CLKs and DYRK1s, respectively. CLKs are implicated in AD pathology by phosphorylating serine residues of arginine-rich (SR) proteins, whereas DYRK1 phosphorylates tau and the cyclic adenosine response element-binding transcription factor (cAMP-CREB). [27,117,118,119,120,121]. DYRK1B has also been linked to ADs, although the main related protein in the family is DYRK1A. DYRK1A has probably not been predicted because there are no molecules similar enough to Meridianin-A in the database used. However, we have found that it can bind to DYRK1B, which is homologous to DYRK1A, but differs in its orthosteric pockets by one amino acid [75,76,77]. It would make sense to study the binding of Meridanin-A to DYRK1A. In relation to phosphorylated Tau kinases, it is also interesting to note that GSK3β (P49841) and CK1δ (P48730) were also predicted as targets (Table S2) but were not among the three top-ranked Meridianin-A complexes, although the energetic differences are small. We should probably think about selecting more high-ranking complexes, or how to make this selection more precise to improve the workflow reported here.

In P15428, HPGD, Meridianin-A binds to the substrate/inhibitor region of P15428, not to the cofactor binding site as Rossinone-A does. As explained above, the inhibitors reported in the literature bind to the substrate binding site sharing interactions with those predicted for Meridianin-A. HPGD is implicated in digital clubbing [122,123], a disorder that is associated with several disorders, such as cardiovascular disease or cancer [124]. The modulators reported in the literature are aimed at inhibiting this enzyme, as HPGD is the key enzyme for the inactivation of prostaglandins, and thus regulates processes such as inflammation or proliferation [92]. Inhibition of HPGD has been designed as a viable strategy for the treatment of various disorders, such as dermal wound healing, bone formation, and hair loss [74,92]. Conversely, down-regulation of HPGD has been associated with increased incidence of several types of cancer and the development of digital clubbing. Loss of enzyme function leads to elevated levels of E2-type prostaglandin, indicating the potential value of HPGD activators in the treatment of cancer and digital clubbing [92,122]. Taken together, this indicates that, although it is possible to target P15428 with Meridianin-A, this MoA is not associated with cardiovascular or neurodegenerative diseases.

Apliacynin-A and Meridianin-A seem to have a tendency of binding to kinases, particularly to Ser/Thr kinases. This tendency is supported by the literature as there are several studies describing these associations [27,75,113,125,126,127]. These molecules contain an indole scaffold that is shared by some kinase inhibitors [128,129,130]. In fact, there are several marine alkaloids sharing a similar structure and putative kinase inhibitory activity [131,132].

Hodgsonal was predicted to interact with P11511, Aromatase (CYP19A1). Aromatase is the enzyme responsible for the final step in the conversion of androgens to the corresponding estrogen. It’s down-regulation has been linked to increased testosterone in autism [133]. However, its main therapeutic implication is in the possible treatment of hormone-dependent breast cancer [53,55]. Hodgsonal binds in the same binding mode as the natural substrate and reported inhibitors, and some of the reported interactions are also established by Hodgsonal. However, for the binding of the natural substrate and the reported inhibitors, the presence of the natural cofactor is important. To better evaluate the binding mode of Hodgsonal with respect to known inhibitors, it should be included in future simulations.

P04798, cytochrome P450 1A1 (CYP1A1), is an enzyme involved in the monooxygenation of structurally diverse compounds ranging from natural products to drugs and prototoxins [85]. CYP1A1 has been related to cardiovascular problems [134,135], but its main therapeutic implication is in cancer [85,87]. Polyrhaphin-A binds within the active site of CYP1A1, but the observed binding mode appears to overlap slightly with the cofactor binding residues, and the cofactor is not included in the simulation. A simulation that includes the cofactor would help to better understand the binding mode and decide whether the Polyrhaphin-A-P04798 complex can still be considered. If Polyhaphin-A is able to bind to the substrate binding region, it could still be considered, but if not, it should be discarded.

P16662, UDP-glucuronosyltransferase 2B7 (UGT2B7), catalyzes the conjugative elimination of drugs as this enzyme is involved in the detoxification of endogenous and exogenous compounds [83,136,137]. It has been implicated in various disorders, mainly cancer [138,139], but also in nerve diseases such as epilepsy [140] and pain [141,142]. This enzyme is able to inactivate, for example, the anticancer drug epirubicin or opioid-based painkillers [83]. A UGT2B7 inhibitor co-administered with drugs that are inactivated by UGT2B7 could allow for smaller doses and fewer side effects [83,143]. Dendrinolide, although showing a reasonably good binding energy when interacting with P16662, is by far the complex with the worst binding energy of the 11 analyzed. Moreover, the predicted binding mode does not correlate with substrate binding, as explained above. This leads us to believe that this complex is not as promising as the others, or at least that the explored binding site is not reliable. In relation to cancer, inhibitors of this enzyme are pursued.

The remaining molecule, Rossinone-A, is related to P15428 and P00352. P15428, HPGD, as explained above, is related to several diseases such as the digital clubbing but especially to cancer. P00352, Aldehyde dehydrogenase 1A1 (ALDH1A1), is involved in the regulation of transcriptional activity, thus in multiple important processes including proliferation, differentiation, and apoptosis. It has been linked to Parkinson’s disease (PD) [144,145], although its main therapeutic indication is cancer. It’s down-regulation increases the risk of PD, so ALDH1A1 activators could be a therapeutic option to combat PD [146]. In contrast, for cancer, their inhibition is being pursued [147,148,149].

For the complexes formed with the two proteins, good results are obtained, and no significant differences are observed between them. This could give rise to possible selectivity problems (especially since both targets share some homology and P00352 has been used as an anti-target of P15428 to test compounds selectivity [92]), although a slight preference for P15428 is observed. Surprisingly, the binding energy obtained for P15428 is the highest and reveals a significant difference (13 kcal/mol) between the binding of Rossinone-A and Meridianin-A, also predicted to interact with it. However, Rossinone-A in complex with P15428 binds to the catalytic site at the interface between the substrate/inhibitor and cofactor binding regions. The dihydroxyphenyl moiety enters the substrate region by orienting the “tail” towards the cofactor binding site, whereas meridianin-A binds to the substrate region. This fact, together with the size difference between the two molecules (larger molecules tend to have higher binding energies in silico), and that the reported inhibitors and activators are expected to bind to the substrate binding site, as commented above, makes further analysis of this complex less interesting, to say the least.

For P00352, the situation is similar. Rossinone-A binds to the cofactor binding region instead of the substrate region. This is logical as both targets are dehydrogenases and in both cases NAD^+^ is the cofactor. Moreover, there is a certain homology between them. Due to the structure of the binding sites and Rossinone-A itself (relatively similar to natural substrates and known inhibitors), a complete binding in the substrate/inhibitors region could be possible. As in the case of Polyrhaphin-A, simulations that include the cofactor would help to better understand the binding mode and decide whether these complexes are worth further analysis.

#### 2.3.4. Complexes Prioritization

According to the results obtained, the most promising complexes are those of Merdianin-A and Aplicyanin-A. In all six complexes, three per molecule, the predicted binding modes coincide with those of the known ligands. In addition, the binding energies obtained showed good values, indicating that the molecule–target interaction could be plausible. However, between Meridianin-A and Apliacynin-A, Meridianin-A should be prioritized as it seems more likely to act within the therapeutic areas of interest, in particular neurodegenerative diseases, while Apliacynin-A seems to be more likely to have an anticancer effect. Among the targets predicted by Meridianin-A, P49759 and Q9Y463 are a more interesting option than P15428, as they are implicated in neurodegenerative disorders, especially AD. In fact, although not initially selected among the first three, Merdianin-A complexes with P49841 and P48730 would be studied before P15428, due to their involvement in the diseases we are interested in.

Hodgsonal can also be selected for further evaluation, although its binding energy is slightly lower, and the observed binding mode is not as good as that of Meridianin-A and Apliacinin-A. This could be a consequence of the fact that P11511, aromatase, has a cofactor whose binding is very important and has not been included in the simulations. In addition, its toxicity profile has been predicted to be less safe than that of Meridianin-A and Apliacinin-A. Finally, Hodgsonal seems to have an anticancer effect rather than against our diseases of interest. Therefore, we place Hodgsonal in a second step.

Complexes with Rossinone-A and Polyrhaphin-A would be in a third step, but before proceeding with any experimental approach, further simulations should be performed, including the cofactor, to check if it can bind to the substrate binding pocket. Based on the observed results and its chemical structure, this does not seem likely to happen in the case of Rossinone-A, but it could happen in the case of Polyrhaphin-A.

Finally, dendrinolide would be in the last step. Similar to what happens with Rossinone-A and Polyrhaphin-A, more simulations are needed. However, in this case, even the binding site targeted by the simulations would have to be rethought. It has become clear that an automatic workflow like ours has not been able to select the correct pocket in this case. The interaction cannot be completely ruled out, as the VP is based on structural similarity to molecules with reported interaction on UDP-glucuronosyltransferase 2B7, so the possibility exists.

## 3. Materials and Methods

### 3.1. Initial Dataset

An initial dataset of 10 compounds was generated from the database owned by Professor Avila. This library is composed of the molecules she has studied and collected over the years. The selection of the initial 10 compounds was done manually, based on their chemical diversity and easy accessibility, to include both chemodiversity and biodiversity representatives.

### 3.2. Virtual Profiling

Cabrakan, two-dimensional (2D), and Hurakan, three dimensional (3D), ligand-based virtual profilings (VP) tools were employed as in [150] or [27]. VP experiments were carried out using marine molecules described in Figure 1 as a seed. Cabrakan uses the Tanimoto coefficient to compare molecules, through the use of 2D (Morgan/circular) fingerprints, over a reference database (Chembl, v19) and the assignment of biological activity. It allows the identification of similar chemical compounds (analogues) to the input molecule. Hurakan compares the query molecule with the structures present in the reference database (Chembl v19) using Comparative Molecular Similarity Indices Analysis (CoMSIA) fields on a 3D grid. Molecules were compared according to the relationship with their environment using the 3D descriptors topologic surface area, lipophilicity, hydrogen bond donors/ acceptors count, and Van der Waals radii, thus obtaining biomimetic compounds with different chemical structures [35,36].

### 3.3. Target Selection

Using DisGENET [37], a database that integrates information on gene–diseases associations, the targets obtained after the VP were related with the pathologies in which they can be involved. Thereafter, we filtered out the targets list, selecting only those related to neurodegenerative and cardiovascular disease.

### 3.4. Target Modelling

3D models of the selected targets were extracted from the Protein Data Bank (RCSB PDB) [151,152]. Then, the protein structures were modelling from them. For those without crystallographic structures available or showing poor sequence representation (<30%), homology models were constructed using SWISS-MODEL [153]. The structures of the marine molecules were modelled from their 2D chemical structure (Figure 2).

### 3.5. Toxicology Prediction

Toxicity was predicted using VEGA-QSAR and the T3DB database [46,154]. VEGA-QSAR, integrating different QSAR models, is able to predict different toxicity endpoints, indicating the reliability of the prediction (ranging from 1 (low reliability) to 3 (high reliability) [46]. To gain statistical significance and reliability, all the available models in VEGA were employed. Because of that, the results of each category were averaged over all the models used and then the results were classified according to its probability of being toxic in the following terms: no toxicity (0), low (<2), medium (2–2.75), or high (2.75–3). To complement the analysis performed with VEGA, the T3DB database was used. A 2D Tanimoto based similarity searching was performed over T3DB containing compounds, using the marine molecules as a seed. If the similarity was >0.65, the marine compounds were classified as toxic, if not as non-toxic [154].

### 3.6. Drug Likeness Evaluation

To evaluate the drug likeness of marine molecules, SuperTarget, a database which provides drug-target relations, was employed [46]. Only those relations that come from the well-known database DrugBank [32] were selected. Later on, the 3D structure of those drugs selected were downloaded from PubChem compound database [47].

### 3.7. Docking Calculations

Docking calculations of the marine molecules were performed using Itzamna and Kin software tools [155,156]. Itzamna is used to carry out docking calculations over crystal structures with co-crystallized ligand(s). It needs the structure of the molecule(s) to dock as well as the protein structure, indicating the ligand ID, as an input. The software automatically calculates the docking box (the region to where the molecules are trying to be docked) over the ligand coordinates. Kin software tool was also used here to perform blind docking calculations over the targets that did not have a co-crystallized ligand or over homology models. Kin involves a cavity searching (and selection of the desired/suitable ones) prior to performing the docking calculation. Different to Itzamna, the docking region (corresponding to some of the found cavities) instead of the ligand ID (then the software automatically calculates the docking box coordinates over it) is given as an input to the calculation, in addition to the molecule to dock and the protein structure. After docking calculations, best binding poses were ranked based on their calculated docking binding energies. The complexes with binding energies lower than −6.5 kcal/mol were selected for further studies.

### 3.8. Molecular Dynamics Simulation

Short (1ns) explicit solvent MD simulations were performed using NAMD v2.11 [157] software tool and took place through four sequential steps: (I) the system was submitted to an energy minimization, followed (II) by a solvent equilibration (using harmonic position restraints on the heavy atoms of the protein–ligand complex) and (III) a slow heating-up phase (0 to 300 K). Finally, (IV) the production simulation was performed. Simulations were carried out using a 2fs time-step and a TIP3P water model. Periodic Boundary conditions were imposed via a cubic box [158]. The distances between the complex and the edge of the box was set to 10 Å. The particle-mesh Ewald method was used to calculate the electrostatic interactions. The temperature and the pressure were kept constant at 300 K and 1 atm, respectively, using Langevin dynamics and a Langevin piston barostat. Bond lengths to hydrogens were constrained with the SHAKE algorithm [159,160]. Before starting the production simulation, all position restraints were removed.

Each protein target was modelled using Amber ff99SB-ILDN and each marine molecule used the General Amber Force Field (GAFF) set of parameters [161,162]. Ligand GAFF parameters were obtained using Antechamber, whereas the protein structures were modelled using the leap module of Amber Tools [163]. 

### 3.9. Molecular Dynamics Analysis

Visual inspection of the trajectories and the analysis of HBs occupancies were performed using Visual Molecular Dynamics (VMD) v1.9.3 [164]. Further thermodynamic (potential, kinetic, and total energy) and structural (Rg, RMSD, and RMSF) analysis were performed using NAMD and GROMACS v2021.3 [165,166].

### 3.10. MM/Generalized Born Surface Area

MM/GBSA rescoring was performed using the MMPBSA python algorithm contained within the Amber Tools suit [163,167]. The snapshots generated at the end of MD simulations were used as input into the post-simulation MM/GBSA calculation of binding free energies [168].

### 3.11. Graphical Representations

Graphical representations of molecule–target complexes were prepared using PyMOL version 2.5 and PLIP version 2.1.8. The graphics are produced using the program Matplotlib and Seaborn python libraries [168,169,170,171]. 2D marine molecules images were prepared using RDKit python library [172].

## 4. Conclusions

In this work, by using different CADD techniques, we have been able to elucidate, from a set of marine molecules from benthic invertebrates, a list of potential targets related to neurodegenerative and cardiovascular pathologies, exemplifying at the same time the therapeutic potential of the marine molecules studied. In general, the therapeutic role of some marine molecules against neurodegenerative diseases like Alzheimer or Parkinson has been recently demonstrated [173,174,175,176,177] as well as in cardiovascular diseases [178,179,180]. All these facts support the results of the study herein presented. Moreover, some of the predictions made here have been validated previously, strongly supporting the proposed pipeline as well as the obtained results [27,125,131]. This fact could even be considered an “experimental validation”. We conducted this study blindly, without any prior information on possible therapeutic applications. Therefore, it is a sign of the usefulness of our approach and an indicator of possible interest in further study of previously unreported results.

From the studied compounds, Meridianin-A and Apliacyanin-A are the most promising. Their predicted complexes seem to be the more plausible. Hodgsonal would be in a next step and the complexes with Rossinone-A, Polyrhaphin-A, and Dendrinolide would be in the last steps. 

Meridianins have been confirmed as kinase inhibitors involved in neurodegenerative diseases and cancer. The predicted targets GSK3β (P49841), CLK1 (P49759), CK1δ (P48730), and DYRK1B (Q9Y463) have been reported in the literature as a possible approach to develop drugs to palliate AD [27,75,125,127,131]. However, it should be noted that GSK3β and CK1δ, although predicted as targets, are not among the top three ranked Meridianin-A complexes, although the differences in binding energy are small. For future studies, it would be interesting to test all the predicted complexes (at least those that reached the MD simulation phase), not just the top three ranked ones. In this sense, it would also be interesting, if necessary, to include cofactors in the simulations, as their presence can improve and clarify the results obtained, making the predictions more robust.

Aplicyanins have also been confirmed to be kinase inhibitors with anticancer activity. In relation to that, many of the studied marine molecules have been proposed as anti-cancer agents [113,114,115]. Consequently, some of the predicted targets, besides being involved in neurodegenerative and cardiovascular diseases, have been reported in the literature as possible targets for potential compounds with anticancer activity. It would be interesting to consider this therapeutic activity in future studies as well.

Regarding the validation of our approach, it is also interesting to note that most of the predicted long-lived HBs and binding modes have been previously reported in the literature as important interactions for ligand binding and/or for proteins to execute their functions. Regarding the predicted long-lived HBs, 87.5% of them (14 out of 16) were previously reported as key binding residues for other compounds.

Although promising starting compounds, marine natural products may present toxicity problems. A preliminary in silico toxicological evaluation has been performed and the results indicated that, in general, the marine molecules studied may present some problems. Further in vitro and in vivo studies are needed to assess potential issues related to toxicity and ADME properties. However, this is not a reason to hinder the therapeutic potential of these compounds, as it is common for the lead optimization phase to include optimization of potency and ADMET properties. In that sense, compounds with toxic profiles, such as the aforementioned Paclitaxel, have arrived in the market.

In terms of methodology, this study is a clear example of how the use of different CADD tools could help to identify potential molecular targets of marine compounds and thus unravel their possible therapeutic potential. The oceans are full of unexplored organisms containing compounds that may have therapeutic potential. Biological and technical difficulties impede the advancement of NPs to pre-clinical stages, but as exemplified in this work, CADD tools can reduce them. Using them, we can identify not only the disease, but also the target and the target cavity/region where the marine molecules bind, prioritizing the most plausible ones. CADD tools also allow us to pinpoint potential toxicity and ADME issues of compounds. This can provide valuable information for further studies, saving time and money to overcome potential problems. All this knowledge can be used in the future to design new drugs.

Finally, a plausible, general, and highly automated workflow for the elucidation of therapeutic agents from marine molecules has been established. This is a process that can be followed in future similar studies. This process can be improved, for example, by adding cofactors to the simulations or changing the number of top-ranked coplexes that are selected, but it is a first step that should be followed by more molecule- and target-specific studies. It is a practical, reasonably quick, and inexpensive way to shed light on potential therapeutic applications of marine natural products that may be very useful in future studies related to drug discovery.

## Figures and Tables

**Table 1:** List of the relevant predicted targets (represented by their Uniprot ID) per molecule by 2D and 3D virtual profiling analysis. Targets with an asterisk are those that have been predicted for more than one marine molecule. UniprotID-protein name association is displayed in Table S1.

Aplicyanin-A	Dendrinolide	Hodgsonal	Meridianin-A	Polyrhaphin-A	Pterenone	Rossinone-A
Q96KQ7 *	P16662	P11511	P49759	P23416	P01375	P83916 *
Q16236 *		P83916 *	Q13627	O75311	P83916 *	Q96KQ7 *
P09874		Q96KQ7 *	Q96KQ7 *	P24046	Q16236 *	Q07343
O15530			Q16236 *	P46098	P15428 *	Q16236 *
P31749			P00374	P14867		P27815
P00491			P48730	P04798		Q08499
			Q13976	P83916		P15428 *
			P49841	Q16236 *		P00352
			P05129			O00255
			Q9Y463			P07550
						Q99714

* Predicted targets for several molecules.

**Table 2:** Toxicology prediction using VEGA software tool for the selected molecules. Results of each category were averaged over all models used and then the results were classified according to their probability of being toxic: no toxicity, low (<2), medium (2–2.75), or high (2.75–3). NO indicates that the reported probability is 0.

Molecule	Carcinogenicity	Mutagenicity	Developmental Toxicity	Skin Sensitization	Average Toxicity
Apliacyanin	LOW	NO	LOW	LOW	LOW
Dendrinolide	LOW	NO	LOW	LOW	LOW
Discorhabdin-B	LOW	LOW	LOW	LOW	LOW
Hodgsonal	MEDIUM	LOW	MEDIUM	HIGH	LOW
Meridianin-A	LOW	LOW	LOW	LOW	LOW
Polyrhaphin-A	MEDIUM	NO	MEDIUM	LOW	LOW
Pteroenone	MEDIUM	LOW	LOW	HIGH	LOW
Rossinone-A	LOW	LOW	LOW	MEDIUM	LOW
Pectinoside-B	LOW	NO	LOW	LOW	LOW

**Table 3:** Predicted HBs that have been reported in the literature. Residue numbers correspond to Wild Type sequence numbering from Uniprot.

Complex	Predicted HBs Reported in the Literature	Literature Reference
Apliacynin-A-O15530	Long-lived: ASP223, LYS111, SER92	[57,58,59,60,61]
Aplicyanin-A-P00491	Long-lived: MET219 Medium-lived: HIS86 Short-lived: ASN243, THR242, GLU201	[64,65,66]
Apliacynin-A-P31749	Long-lived: SER205 Medium-lived: ASP242 Short-lived: TRP80	[62,63]
Meridianin-A-P15428	Long-lived: GLN148 Medium-lived: SER138 Short-lived: GLU184	[71,72,73,74]
Meridianin-A-P49759	Long-lived: Glu242 Medium-lived: LYS191 Short-lived: LEU244	[80,90,91]
Meridianin-A-Q9Y463	Long-lived: LYS140, GLU191	[75,76,77]
Rossinone-A-P15428	Long-lived: ASN91 Medium-lived: GLN148, ILE17, GLN15 Short-lived: TYR151, GLY93, VAL186, GLY12	[71,72,73,74]
Rossinone-A-P00352	Long-lived: GLU196, GLU269, GLU400 Medium-lived: LYS193	[70]
Hodgsonal-P11511	Long-lived: MET374 Short-lived: LEU477	[53,54,55,56]
Dendrinolide-P16662	Medium-lived: TRP356	[83]
Polyrhaphin-A-P04798	Short-lived: ILE386, SER322	[84,85,86,87]

## Data Availability

Not applicable.

## Supplementary Materials

The following supporting information can be downloaded at: https://www.mdpi.com/article/10.3390/md20010053/s1. Table S1. UniprotID–Protein name–Gene name correspondence of the targets selected after VP experiments; Table S2. Summary of the results obtained performing docking simulations and Molecular Mechanics/Generalized Born Surface Area (MM/GBSA) calculations of marine molecules against each target (UniProt) with crystallographic structures (PDB) with ligand; Table S3. Summary of the results obtained performing blind docking simulations and Molecular Mechanics/Generalized Born Surface Area (MM/GBSA) calculations of marine molecules against each target (UniProt) represented by homology models; Table S4. Summary of the results obtained performing blind docking simulations and Molecular Mechanics/Generalized Born Surface Area (MM/GBSA) calculations of marine molecules against each target (UniProt) represented by crystallographic structures (PDB) without ligand; Table S5. Summary of the best affinities obtained after Molecular Mechanics/Generalised Born Surface (MM/GBSA) calculations of the marine molecules against each target (UniProt) and, in addition, the pathologies per target are listed. MD MM/GBSA correspond to the calculation after the MD using the trajectory generated as an input. Docking MM/GBSA correspond to the first frame of the trajectory (docking pose embedded into a water box and then the solvated docking complex minimized and equilibrated); Table S6. Summary of the interactions found after docking and Molecular Dynamics simulations. Images of the binding mode of each marine molecule inside the binding cavity of the corresponding target (corresponding to the best docking pose and last frame of the MD trajectory, respectively); Table S7. Summary of the binding energies per each target and structure from drugs and marine molecule–target. Figure S1. 2D Tanimoto based similarity results, where each column represents the maximum similarity found per molecule; Figure S2. The graphics show the potential energy, the kinetic energy, and the total energy during the progress of the MD simulation of the eleven systems; Figure S3. The graphics show the Radius of gyration (Rg) of the MD simulation of the eleven systems; Figure S4. Root-mean-square deviation (RMSD) values of each target along the corresponding target–ligand molecular dynamics (MD) simulation; Figure S5. Root-mean-square deviation (RMSD) values of each ligand along the corresponding target–ligand molecular dynamics (MD) simulation; Figure S6. Root-mean-square deviation (RMSD) values of each target–ligand complex along the molecular dynamics (MD) simulation; Figure S7. Root-mean-square fluctuation (RMSF) per residue (X-axis) values of each system along the MD simulation.
