# Supplementary material for: Computer-Aided Drug Design (CADD) to De-Orphanize Marine Molecules: Finding Potential Therapeutic Agents for Neurodegenerative and Cardiovascular Diseases
Source: Mar Drugs. 2022 Jan 5;20(1):53. doi: 10.3390/md20010053 (PMC8781171; doi:10.3390/md20010053)
Supplement: Supplementary file 1 [file marinedrugs-20-00053-s001.zip › marinedrugs-1527792-supp.pdf]

Supplementary Materials

Table S1. UniprotID–Protein name–Gene name correspondence of the targets selected after VP experiments (Table 1). In bold there are the selected targets (10) after the in silico binding studies. 1 of them, P15428, was selected twice, for two different compounds. Thus, 11 molecule–target complexes were finally selected corresponding to 10 targets.

UniProt ID	Protein name	Gene name
Q96KQ7	Histone-lysine N-methyltransferase (EHMT2)	EHMT2
Q16236	Nuclear factor erythroid 2-related factor 2 (NF-E2-related factor 2)	NFE2L2
P09874	Poly [ADP-ribose] polymerase 1 (PARP-1)	PARP1
**O15530**	**3-phosphoinositide-dependent protein kinase 1 (hPDK1)**	**PDK1**
**P31749**	**RAC-alpha serine/threonine-protein kinase**	**AKT1 PKB**
**P00491**	**Purine nucleoside phosphorylase (PNP)**	**PNP**
**P16662**	**UDP-glucuronosyltransferase 2B7 (UGT2B7)**	**UGT2B7**
**P11511**	**Aromatase**	**CYP19A1**
P83916	Chromobox protein homolog 1 (HP1Hsbeta)	CBX1
**P49759**	**Dual specificity protein kinase (CLK1)**	**CLK1**
Q13627	Dual specificity tyrosine-phosphorylation-regulated kinase 1A (hMNB)	DYRK1A
**Q9Y463**	**Dual specificity tyrosine-phosphorylation-regulated kinase 1B (Mirk protein kinase)**	**DYRK1B**
P00374	Dihydrofolate reductase	DHFR
P48730	Casein kinase I isoform delta (CKI-delta)	CSNK1D HCKID
Q13976	cGMP-dependent protein kinase 1 (cGK1)	PRKG1
P49841	Glycogen synthase kinase-3 beta (GSK-3 beta)	GSK3B
P05129	Protein kinase C gamma type (PKC-gamma)	PKCG
P23416	Glycine receptor subunit alpha-2	GLRA2
O75311	Glycine receptor subunit alpha-3	GLRA3
P24046	Gamma-aminobutyric acid receptor subunit rho-1 (GABA(A) receptor subunit rho-1)	GABRR1
P46098	5-hydroxytryptamine receptor 3A (5-HT3A)	5HT3R
P14867	Gamma-aminobutyric acid receptor subunit alpha-1 (GABA(A) receptor subunit alpha-1)	GABRA1
**P04798**	**Cytochrome P450 1A1 (CYPIA1)**	**CYP1A1**
P01375	Tumor necrosis factor (Cachectin) (TNF-alpha)	TNFA TNFSF2
**P15428**	**15-hydroxyprostaglandin dehydrogenase [NAD(+)] (15-PGDH)**	**PGDH1**
Q07343	cAMP-specific 3′5′-cyclic phosphodiesterase 4B	PDE4B
P27815	cAMP-specific 3′5′-cyclic phosphodiesterase 4A	PDE4A
Q08499	CAMP-specific 3′5′-cyclic phosphodiesterase 4D	PDE4D
**P00352**	**Retinal dehydrogenase 1 (RALDH 1)**	**ALDH1**
O00255	Menin	MEN1
P07550	Beta-2 adrenergic receptor (Beta-2 adrenoreceptor)	ADRB2
Q99714	3-hydroxyacyl-CoA dehydrogenase type-2	HADH2

Table S2. Summary of the results obtained performing docking simulations and Molecular Mechanics/Generalized Born Surface Area (MM/GBSA) calculations of marine molecules against each target (UniProt) with crystallographic structures (PDB) with ligand. To avoid false positive, each docking calculation was performed twice (R0/R1). All the energy values are in kcal/mol.

UniProt	PDB - Ligand	Docking	MM/GBSA	UniProt	PDB - Ligand	Docking	MM/GBSA
		Binding Energy	Binding Energy			Binding Energy	Binding Energy
		R0/R1				R0/R1	
	**Hodgsonal**				**Aplicyanin-A**		
**P11511**	3EQM-ASD	-8.1 / -8.2	-34.1795	**P09874**	1UK0-FRM	-8.2 / -8.2	-32.3788
	3EQM-HEM	-8.3 / -8.4	-22.4488	**O15530**	2R7B-253	-7.7 / -7.7	-22.8350
**P15428**	2GDZ-NAD	-6.9 / -6.9	-6.3093		3QC4-MP7	-8.1 / -8.1	-52.7520
**Q99714**	1U7T-NAD	-7.2 / -7.2	-0.0562	**P31749**	3O96-IQO	-8.6 / -8.6	-43.5072
	2O23-NAD	-6.7 / -6.7	-14.0999	**P00491**	1ULB-GUN	-7.2 / -7.2	-45.5407
	**Meridianin-A**				**Rossinone-A**		
**P00374**	1MVS-DTM	-7.7 / -7.7	-28.9913	**P00352**	4WB9-NAI	-8.8 / -8.5	-48.1930
**P48730**	4KBK-1QG	-7.3 / -7.2	-34.4325	**O00255**	3U88-CHD	-6.8 / -6.8	-25.5673
**Q13976**	3OGJ-CMP	-6.7 / -6.7	-28.4003	**P07550**	4GBR-CAU	-9.7 / -9.7	-43.2079
	4QX5-CMP	-7.9 / -7.9	-13.5921	**Q99714**	1U7T-TDT	-8.9 / -9	-39.1248
**P49841**	3PUP-OS1	-7.7 / -7.7	-29.1106		1U7T-NAD	-8.1 / -8	-40.7381
**Q99714**	1U7T-TDT	-7.7 / -7.7	-25.4862		2O23-NAD	-8.6 / -8.5	-39.0733
	1U7T-NAD	-7.5 / -7.4	-21.3118	**P15428**	2GDZ-NAD	-9.2 / -9.3	-51.7908
	2O23-NAD	-7.6 / -7.6	-23.5035	**P04637**	5AB9-92O	-6.4 / -6.5	-31.0615
**Q13627**	4AZE-3RA	-8.2 / -8.2	-32.8447				
**P15428**	2GDZ-NAD	-8.4 / -8.4	-38.3083				
	**Pteroenone**						
**P15428**	2GDZ-NAD	-7 / -7.1	-30.7877				
**P07550**	4GBR-CAU	-7.7 / -7.6	-36.9769				
**Q99714**	1U7T-TDT	-6.5 / -6.5	-23.3863				
	2O23-NAD	-6.5 / -6.4	-22.0587				

Table S3. Summary of the results obtained performing blind docking simulations and Molecular Mechanics/Generalized Born Surface Area (MM/GBSA) calculations of marine molecules against each target (UniProt) represented by homology models. Pocket means the cavity chosen to perform MD simulations. To avoid false positives, each docking calculation was performed twice (R0/R1). All the energy values are in kcal/mol.

UniProt	Pocket	Docking	MM/GBSA	UniProt	Pocket	Docking	MM/GBSA
		Binding Energy	Binding Energy			Binding Energy	Binding Energy
		R0/R1				R0/R1	
		**Hodgsonal**				**Rossinone-A**	
**Q96KQ7**	0	-7.8 / -7.9	-20.4079	**Q96KQ7**	1	-8.1 / -8.1	-26.9966
		**Meridianin-A**		**Q07343**	0	-8.6 / -8.6	-41.0540
**P49759**	0	-9.3 / -9.3	-37.9952	**Q16236**	0	-8.1 / -8	-36.1075
**Q96KQ7**	0	-7.3 / -7.3	-19.5406	**P27815**	0	-8.8 / -8.7	-37.3115
**Q9Y463**	0	-8.1/ -8.4	-37.6179	**Q08499**	0	-9.2 / -9.3	-32.3433
		**Pteroenone**				**Polyrhaphin-A**	
**Q16236**	0	-6.2 / -6.5	-15.8940	**P24046**	0	-7.5 / -7.6	-27.6724
		**Aplicyanin-A**		**P46098**	1	-8.1 / -8.1	-30.8251
**Q96KQ7**	0	-7.5 / -7.5	-21.8578	**P14867**	0	-7.6 / -7.5	-14.0650
**Q16236**	0	-6.6 / -6.6	-22.3084	**P04798**	3	-9.5 / -9.2	-42.6268

Table S4. Summary of the results obtained performing blind docking simulations and Molecular Mechanics/Generalized Born Surface Area (MM/GBSA) calculations of marine molecules against each target (UniProt) represented by crystallographic structures (PDB) without ligand. Pocket means the cavity chosen to perform MD simulations. To avoid false positive, each docking calculation was performed twice (R0/R1). All the energy values are in kcal/mol**.**

UniProt	PDB	Pocket	Docking	MM/GBSA	UniProt	PDB	Pocket	Docking	MM/GBSA
			Binding Energy	Binding Energy				Binding Energy	Binding Energy
			R0/R1					R0/R1	
		**Hodgsonal**					**Rossinone-A**		
**P04637**	3Q01	1	-6.1 / -6.6	-11.3246	**P04637**	3Q01	1	-7 / -6.5	-23.5341
		**Meridianin-A**					**Dendrinolide**		
**Q13976**	4KU8	0	-7.1 / -7.1	-19.9742	**P16662**	2O6L	0	-8.1 / -8.1	-25.6552

Table S5. Summary of the best affinities obtained after Molecular Mechanics/Generalised Born Surface (MM/GBSA) calculations of the marine molecules against each target (UniProt) and, in addition, the pathologies per target are listed. MD MM/GBSA correspond to the calculation after the MD using the trajectory generated as an input. Docking MM/GBSA correspond to the first frame of the trajectory (docking pose embedded into a water box and then the solvated docking complex minimized and equilibrated). The pathologies listed correspond to the entry with the most PMID references found in DisGeNet for the therapeutic areas of interest: Neurodegenerative and cardiovascular diseases (Digital clubbing was selected because it is associated with a number of diseases, mostly of the heart and lungs). Each target can be represented by crystallographic structures with ligands, without ligands or homology models (HM). To avoid false positives, each docking calculation was performed twice (R0/R1). All energy values are in kcal/mol.

UniProt	PDB-Ligand	Docking	Docking MM/GBSA	MD MM/GBSA	Pathologies
		Binding Energy	Binding Energy	Binding Energy	
			**Hodgsonal**		
**P11511**	3EQM-ASD	-8.1 / -8.2	-27.4701	-34.1795	Autism
			**Meridianin-A**		
**P15428**	2GDZ-NAD	-8.4 / -8.4	-40.9594	-38.3083	Digital clubbing
**P49759**	HM	-9.3 / -9.3	-41.2008	-37.9952	Alzheimer
**Q9Y463**	HM	-8.1 / -8.4	-36.7694	-37.6179	Alzheimer
			**Aplicyanin-A**		
**O15530**	3QC4-MP7	-8.1 / -8.1	-54.9308	-52.7520	Heart failure
**P00491**	1ULB-GUN	-7.2 / -7.2	-59.6093	-45.5407	Alzheimer
**P31749**	3O96-IQO	-8.6 / -8.6	-56.5745	-43.5072	Schizophrenia
			**Rossinone-A**		
**P15428**	2GDZ-NAD	-9.2 / -9.3	-50.9703	-51.7908	Digital clubbing
**P00352**	4WB9-NAI	-8.5 / -8.8	-45.2846	-48.1930	Parkinson
			**Polyrhaphin-A**		
**P04798**	HM	-9.2 / -9.5	-40.3382	-42.6268	Cardiovascular disease
			**Dendrinolide**		
**P16662**	2O6L	-8.1 / -8.1	-31.8563	-25.6552	Epilepsy

Table S6. Summary of the interactions found after docking and Molecular Dynamics simulations. Images of the binding mode of each marine molecule inside the binding cavity of the corresponding target (corresponding to the best docking pose and last frame of the MD trajectory, respectively). Marine molecules and interacting residues are represented in sticks. Orange lines indicate HBs, gray dashed lines hydrophobic interactions, green dashed lines. HB=Hydrogen bond; HI=Hydrophobic interaction; PS=π-stacking; SB=Salt Bridge. After the residue number there is .A or .B because this protein is a dimer and indicates the chain to which the residue belongs. Higher resolution images of the binding modes can be found at https://zenodo.org/record/5707582.

**Complex**	**Docking**		**Molecular Dynamics**	
	Interactions List	Binding mode	Interactions List	Binding Mode
Apliacynin-A<br>O15530	HI LYS111<br>HI VAL143<br>HI LEU159<br>HI ASP223<br>PS TYR126	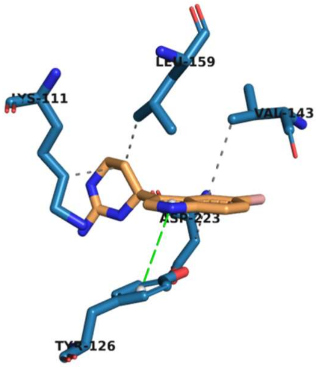	HI TYR126<br>HI VAL143<br>HI ASP223<br>HB SER92<br>HB LYS111<br>HB TYR126<br>HB ASP223	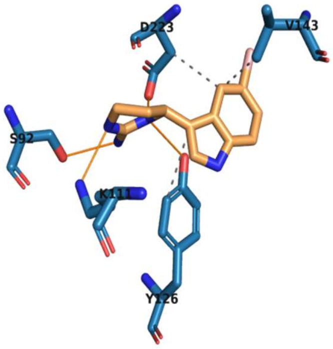
Aplicyanin-A<br>P00491	HI PHE200<br>HI PHE200<br>HB ALA116<br>HB ASN243	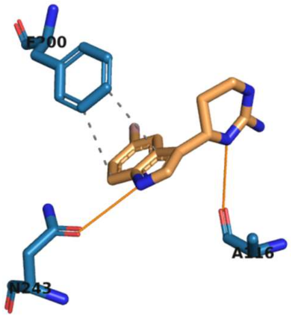	HB HIS86<br>HB ALA116<br>HB ALA116<br>HB MET219<br>HB THR242	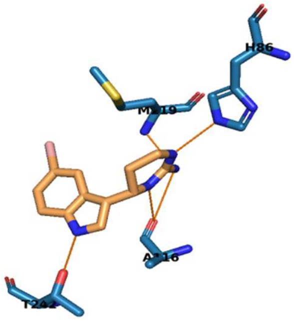
Apliacynin-A<br>P31749	HI TRP80<br>HI TRP80<br>HI LEU210<br>HI LEU264<br>HI LEU264<br>HI LYS268<br>HB THR211<br>HB ILE290<br>PS TRP80<br>PS TRP80	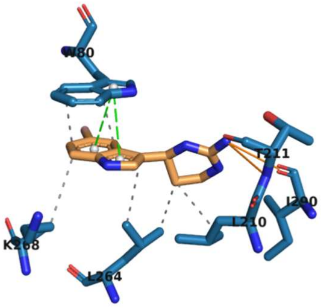	HI TRP80<br>HI TRP80<br>HI TYR263<br>HI LYS268<br>HB SER205<br>HB THR211<br>HB ILE290<br>HB ASP292	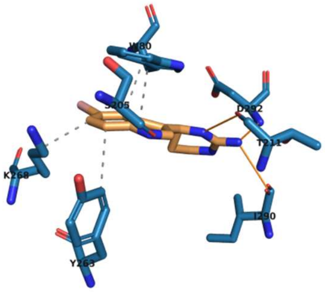

Meridianin-A<br>P15428	HI VAL145<br>HI PHE185<br>HI PHE185<br>HB SER137	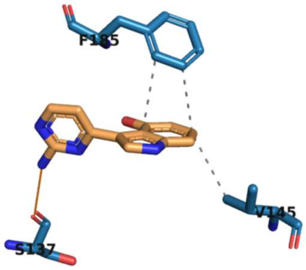	HI ALA140<br>HI VAL145<br>HI TYR151<br>HI PHE185<br>HI PHE185<br>HI ILE190<br>HI LEU191<br>HB SER138<br>HB LEU139<br>HB ALA140<br>HB GLY184<br>HB VAL186	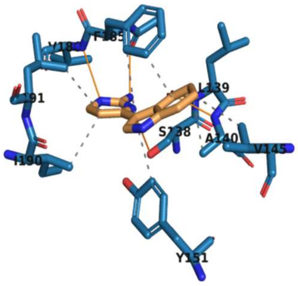
Meridianin-A<br>P49759	HI LEU167<br>HI LEU167<br>HI VAL175<br>HI ALA189<br>HI VAL225<br>HI PHE241<br>HI LEU244<br>HI LEU295<br>HI VAL324<br>HB LYS191<br>HB ASP325	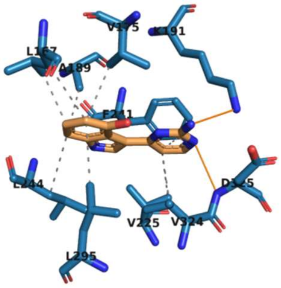	HI LEU167<br>HI ALA189<br>HI PHE241<br>HI LEU244<br>HB LYS191<br>HB GLU242<br>HB ASP325<br>HB ASP325	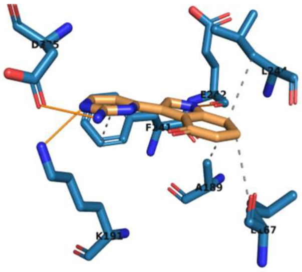
Meridianin-A<br>Q9Y463	HI ILE117<br>HI VAL174<br>HI LEU193<br>HI LEU246<br>HI VAL258<br>HI VAL258<br>HB LYS140<br>HB ASP259<br>PS PHE190	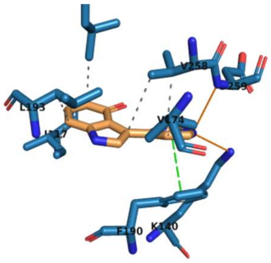	HI ILE117<br>HI ALA138<br>HI VAL174<br>HI PHE190<br>HI LEU246<br>HI VAL258<br>HB LYS140<br>HB GLU106<br>HB ASP174	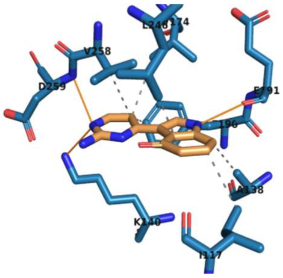
Rossinone-A<br>P15428	HI GLN15<br>HI PRO183<br>HI PHE185<br>HI ILE190<br>HI LEU191<br>HB ILE17<br>HB GLY18<br>HB ASN91	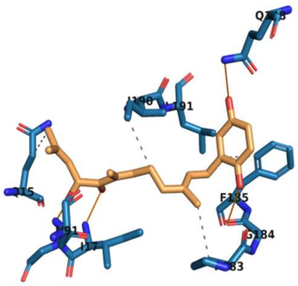	HI GLN15<br>HI VAL94<br>HI ALA140<br>HI TYR151<br>HI PHE185<br>HI VAL186<br>HI ILE190<br>HI ILE190	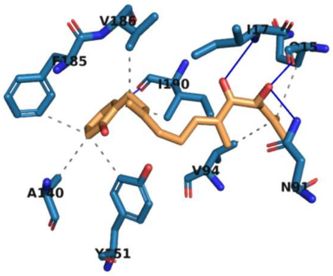

	HB GLN148		HB GLN15	
	HB GLY184		HB ILE17	
			HB ASN91	
			HB ILE190	
Rossinone-A	HI ILE166	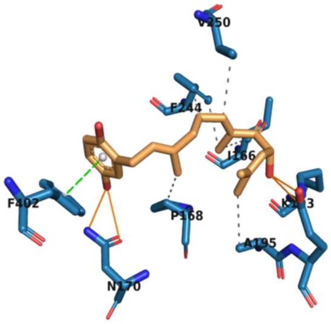	HI ILE166	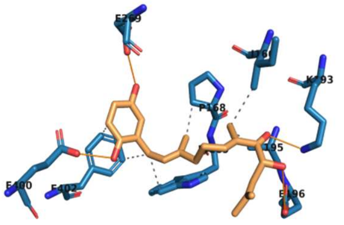
P00352	HI PRO168		HI PRO168	
	HI ALA195		HI TRP169	
	HI PHE244		HI ALA195	
	HI PHE244		HI PHE402	
	HI VAL250		HI PHE402	
	HB ASN170		HI PHE402	
	HB ASN170		HB LYS193	
	HB LYS193		HB GLU196	
	HB GLU196		HB GLU269	
	PS PHE402		HB GLU400	
Hodgsonal	HI ILE133	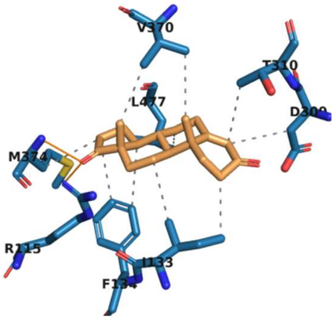	HI ILE133	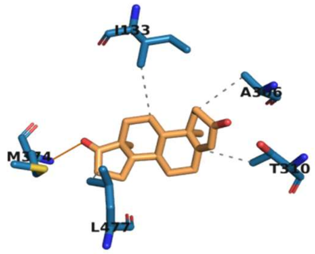
P11511	HI ILE133		HI ALA306	
	HI PHE134		HI THR310	
	HI PHE134		HI LEU477	
	HI ASP309		HB MET374	
	HI THR310			
	HI VAL370			
	HI VAL370			
	HI MET374			
	HI LEU477			
	HI LEU477			
	HB ARG115			
	HB MET374			
Dendrinolide	HI PRO289.A	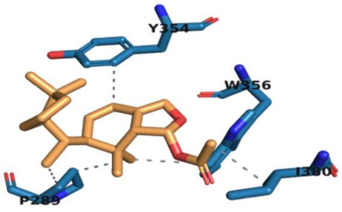	HI LYS355.A	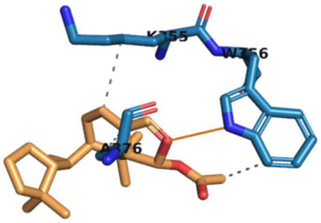
P16662	HI PRO289.A		HI TRP356.A	
	HI TYR354.A		HI ALA376.B	
	HI TRP356.A		HB TRP356.A	
	HI ILE380.B			

Polyrhaphin-A<br>P04798	HI ASP313<br>HI LEU314<br>HI VAL382<br>HI ILE386<br>HI ILE386<br>HI LEU496<br>HI LEU496<br>HB ILE386<br>SB ARG106	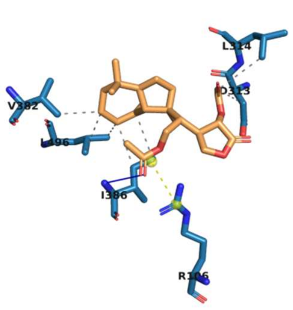	HI PHE123<br>HI ILE198<br>HI ALA317<br>HI ILE386<br>HI PHE450<br>HI ILE458<br>HB ARG106<br>HB ILE458	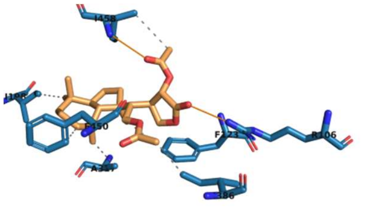

Structure-based drug-likeness evaluation

To pre-validate the viability of the resulting molecule–target complexes from the VP experiment, we checked if the molecules could behave as other known drugs for the predicted targets. To evaluate if the marine molecules share a similar behavior with known drugs regarding their (docking) binding energy, per each of the targets found on the VP, a list of known drugs were retrieved, from each of the targets, from Drugbank. A total of 190 drugs related with 17 of the 32 targets (which represents five of the seven marine molecules) were obtained. Docking calculations with each drug–target and marine molecule–target were performed against crystallographic structures or homology models, depending on the case (Table S7).

Table S7. Summary of the binding energies per each target and structure from drugs and marine molecule–target. Drugs binding energies are an average of all the docking energies per target and structure. To avoid false positives, each docking calculation was performed twice (R0/R1). Energy values are expressed on kcal/mol. Drug–target complexes with energy values lower than -6.5 kcal/mol (based on the bibliography and previous experience it is a reasonable threshold to avoid false positives) were discarded. 14 complex in total. The remaining energies, if there were more than one, were averaged by target and structure. PDB-LIGAND means the PDB ID of the employed structure and the cavity (generated from the coordinates of the mentioned co-crystal). HM refers to a homology model over a blind docking was carried out. It means a cavity searching was done prior to the docking calculation.

	**PDB - LIGAND**	**DRUGS FOUND**	**DRUGS**	**MOLECULE**	
			Binding Energy R0/R1	Binding Energy R0/R1	
**P11511**	3EQM-ASD	5	-7,5 / -7,6	-8,1 / -82	Hodgsonal
	3EQM-HEM	5	-8,4 / -8,4	-8,3 / -8,4	Hodgsonal
**P04798**	1UK0-FRM	3	-10,1 / -10,1	-8,2 / -8,2	Polyrhaphin-A
**O15530**	2R7B-253	4	-10 / -10	-7,7 / -7,7	Apliacyanin
	3QC4-MP7	4	-7,5 / -7,5	-8,1 / -7,6	Apliacyanin
**P31749**	3O96-IQO	2	-9 / -9	-8,6 / -8,6	Apliacyanin
**P00491**	1ULB-GUN	7	-7,5 / -7,5	-7,2 / -7,2	Apliacyanin
**P15428**	2GDZ-NAD	1	-11,9 / -11,8	-7,9 / -7,9	Meridianin A
**P00374**	1MVS-DTM	15	-8,9 / -8,9	-7,7 / -7,7	Meridianin A
**P49841**	3PUP-OS1	6	-9,2 / -9,4	-7,7 / -7,7	Meridianin A
**P00352**	4WB9-NAI	3	-8,4 / -8,5	-8,8 / -8,5	Rossinone
**P07550**	4GBR-CAU	33	-8 / -8,1	-8,7 / -8,7	Rossinone
**Q99714**	1U7T-NAD	1	-10,1 / -10,1	-7,6 / -7,6	Rossinone
	1U7T-TDT	1	-10,7 / -10,7	-7,7 / -7,7	Rossinone
**Q07343**	HM	16	-7,2 / -7,2	-8,6 / -8,6	Rossinone

**P14867**	HM	46	-6,7 / -6,8	-7,6 / -7,4	Polyrhaphin-A
**P24046**	HM	1	-7,9 / -7,9	-7,5 / -7,6	Polyrhaphin-A
**P27815**	HM	10	-7,3 / -7,3	-8,8 / -8,7	Rossinone
**P46098**	HM	10	-8,4 / -8,4	-8,1 / -8,1	Polyrhaphin-A
**Q08499**	HM	3	-8 / -8	-9,2 / -9,1	Rossinone

The obtained results (see Table S7) show up a maximum difference of ±3 kcal/mol between drug-target and marine molecule–target in those represented by crystallographic structures, and ±2 kcal/mol for those represented by homology models. These results allow us to suggest that the studied marine molecules could behave, or at least display, a binding strength, similar to known drugs.

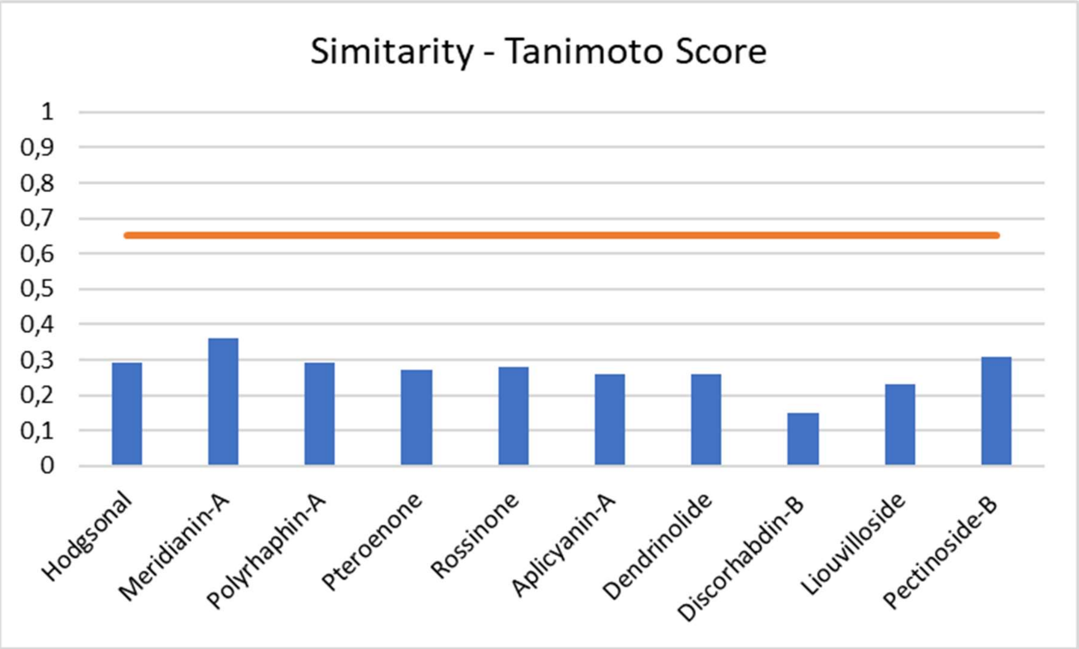

Figure S1. 2D Tanimoto based similarity results, where each column represents the maximum similarity found per molecule. The orange line represents the Tanimoto score, 0.65, from which it is considered that two molecules are similar.

Energy analysis

Potential Energy of the protein–ligand complexes, as well as kinetic and total energies, were analyzed from NAMD simulation outputs, showing that all the simulations were stable.

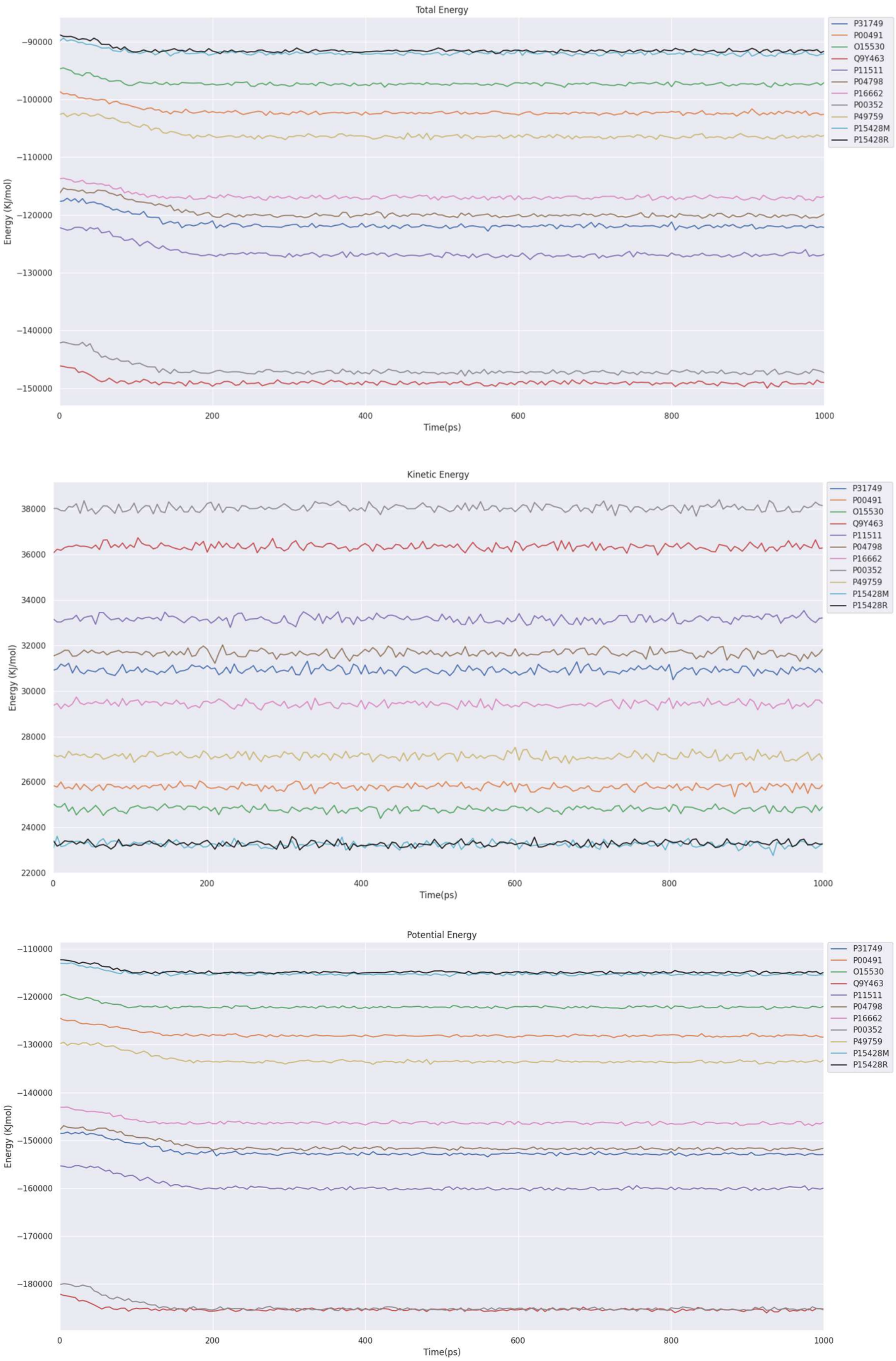

Figure S2. The graphics show the potential energy, the kinetic energy, and the total energy during the progress of the MD simulation of the eleven systems. The color code of each target can be seen in the legend box. P15428M makes reference to the complex with Meridianin-A and P15428R with Rossinone-A.

Rg analysis

The analysis of the radius of gyration (Rg), which allows to analyze the compactness of the protein and is related to the tertiary structure of the studied targets, was carried out on the target–ligand molecular dynamics simulations. Changes in Rg values are an indicator of conformational transitions in the protein. In this study, all proteins appear to be stable in the presence of a ligand. However, the simulations performed, while long enough for their original purpose, are probably too short to fully assess the behavior of Rg. These results should probably be considered a first look at Rg's behavior.

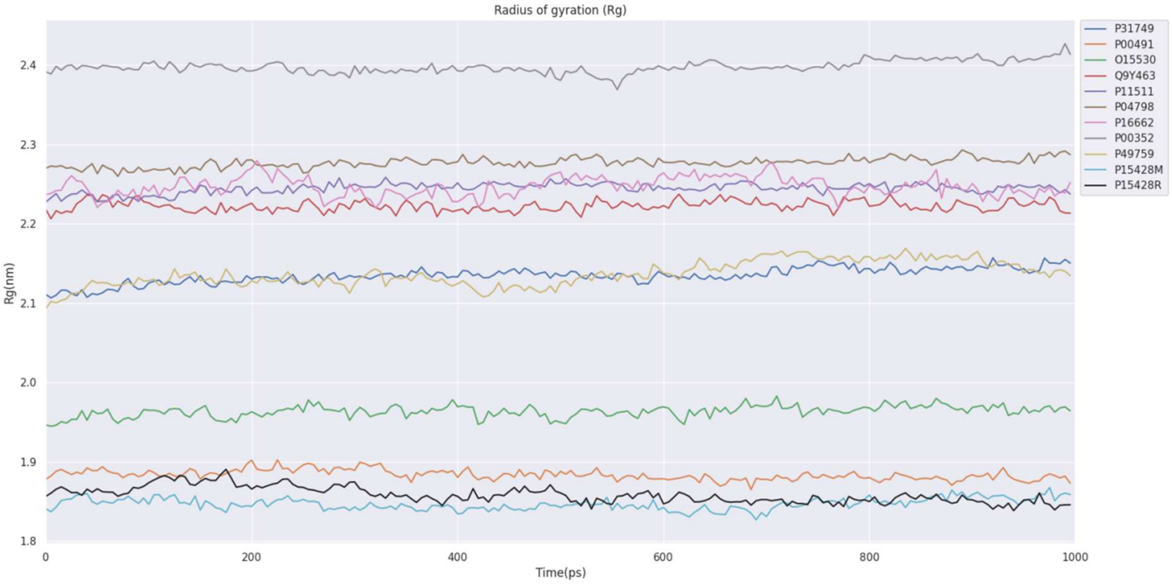

Figure S3. The graphics show the Radius of gyration (Rg) of the MD simulation of the eleven systems. The color code of each target can be seen in the legend box. P15428M makes reference to the complex with Meridianin-A and P15428R with Rossinone-A.

Root-mean-square deviation (RMSD) analysis

RMSD analysis of the protein (Figute S4), the ligand (Figure S5), and the protein–ligand complex (Figure S6) during MD simulations were performed. RMSD is used to validate the stability along the simulations and measures the average distance between the atoms of superimposed structures extracted from the MD simulations and a reference structure (form the first/initial frame of the generated trajectory in this case). Targets and Target–Ligand complexes are very stable. Ligands are also stable, which correlate with the fact that they do not leave the binding site. Interestingly, it can be observed that Dendrinolide (P16662) and Polyrhaphin-A (P04798), for which no long-lived HB was found, show the highest RMSD values followed by Rossinone-A (P00352 and P15428), which binds to cofactor binding region, instead to the substrate binding region, in both cases as Polyrhapin-A also does.

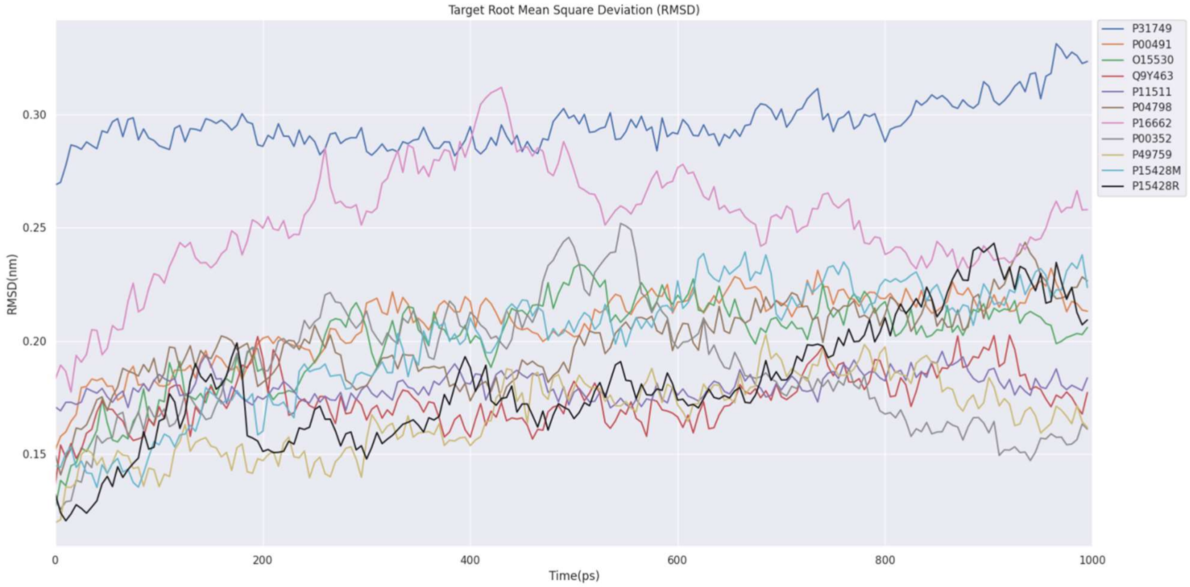

Figure S4. Root-mean-square deviation (RMSD) values of each target along the corresponding target–ligand molecular dynamics (MD) simulation. The color code for each system can be seen in the legend box. P15428M makes reference to the complex with Meridianin-A and P15428R with Rossinone-A.

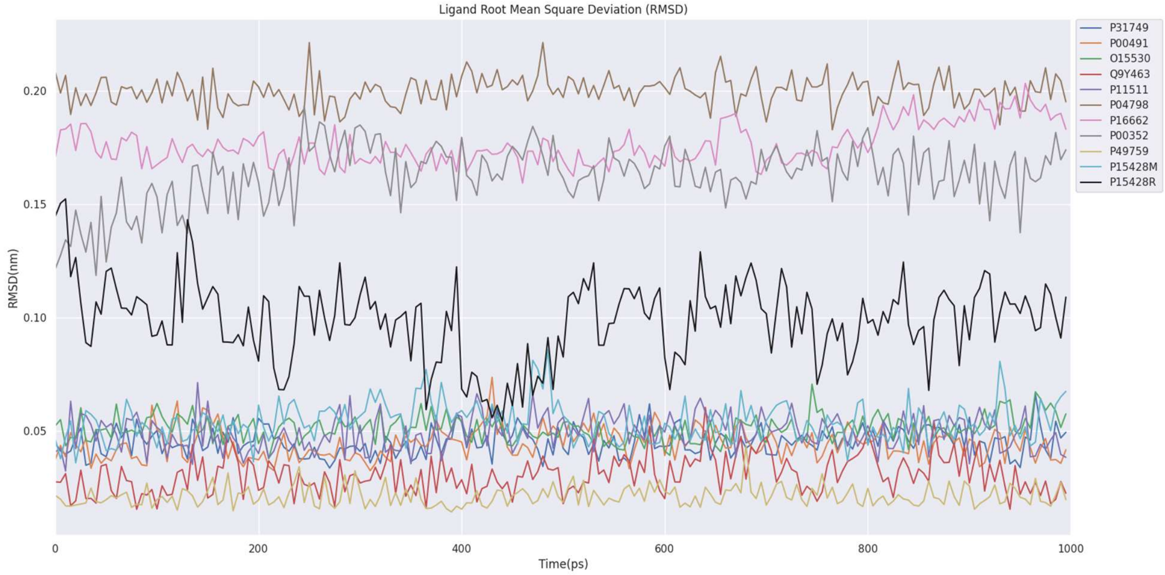

Figure S5. Root-mean-square deviation (RMSD) values of each ligand along the corresponding target–ligand molecular dynamics (MD) simulation. The color code for each system can be seen in the legend box. P15428M makes reference to the complex with Meridianin-A and P15428R with Rossinone-A.

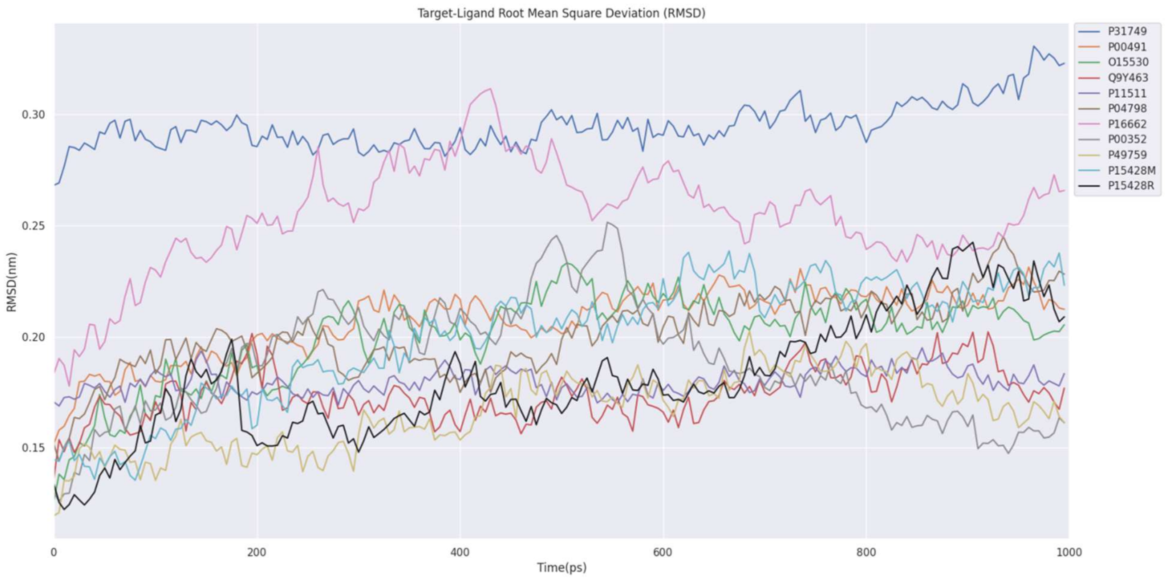

Figure S6. Root-mean-square deviation (RMSD) values of each target–ligand complex along the molecular dynamics (MD) simulation. The color code for each system can be seen in the legend box. P15428M makes reference to the complex with Meridianin-A and P15428R with Rossinone-A.

Root-mean-square fluctuation (RMSF) analysis

Another hallmark of protein stability is RMSF. When a dynamical system such as a protein fluctuates about some well-defined average position, the RMSF, amplitude of atomic motions, can be calculated from the MD trajectory (Figure S7). This amplitude reflects the flexibility of individual or sets of atoms.

RMSFs values higher than 0.25 nm, highlighted in orange on Figure S7, are characteristic of amino acid residues belonging to flexible regions. For the 11 systems analyzed here, all of them display RMSFs values higher than 0.25 nm. As a general fact, most of the higher peaks are located in the N and C terminal regions of the simulated protein sequences, which can be normal due to the high flexibility of protein terminal regions but also can be related with the resolution/quality of the employed structure.

Focusing on each particular case, Q9Y463 and P11511 do not show high flexible regions. They only have a small single peak in the Nterm region on the modeled sequences. The situation is similar for P15428, complexed with Meridianin-A and Rossinone-A, which only shows a peak in the Nterm region. In these cases, the peaks are wider and involve more residues than for Q9Y643 and P11511. The observed fluctuations are higher for the complex with Rossinone-A than with Meridianin-A. It could be a sign of Rossinone-A-P15428 binding being less stable than with Meridianin-A, and it can be also related to the higher RMSD observed for Rossinone-A (Figure S5).

P49759 has fluctuations with values higher than 0.3 nm between residues 161-163 (308-310 WT) surrounded by other residues with near values. This system has another point of small fluctuations around residue 191 (338 WT), and another single peak in the Nterm region of the modeled protein sequence.

O15530 seems to be a rather rigid system, even though before the highest fluctuation observed on residues 54-56 (129-131 WT), two other lower peaks can be observed.

P00491 show fluctuations in the Nterm region, single peak, and a higher and wider peak in the Cterm region. Moreover, small fluctuations can be observed between residues 252 and 253 (same numbering for the WT sequence).

P31749 has several single fluctuation peaks. They are surrounded by several residues with near, but lower, values. Residues 1(5 WT), 2 (6 WT), 103 (107 WT), and 292 (296 WT) show RMSF values higher than 0.25 nm. There can be other fluctuating regions found, like around residue 50, 150, or 200, but with values lower than 0.25 nm.

P00352 results reveals the existence of flexible regions, as there are two high (> 0.4 nm) peaks surrounded by residues with lower values, but some of them have a RMSF higher than 0.25 nm. These are located around residues 139 to 144 (147 to 151 WT) and in the Cterm region of the modeled sequence around residues 486 and 493 (494 and 501 WT).

P04798 has two fluctuating regions around residues 261-262 (296-297 WT) and 452 (487 WT), despite the previous residues between 230 (265 WT) and 245 (270 WT) also showing some lower fluctuation.

P16662 shows three peaks (>0.25 nm) at residues 118 (135 WT), 119 (136 WT), 155 (172 WT), 164 (181 WT) surrounding residues with near, but lower, values conforming a quite wide flexible region.

These results are in line with the observed HBs' behavior. The residues involved in the molecule binding, especially those found with long lived HBs occupancies, are placed on the rigid regions of the proteins. However, some identified binding residues (Table S6) are near some of the identified flexible regions. In any case, it seems that the most flexible regions do not affect the marine molecule binding.

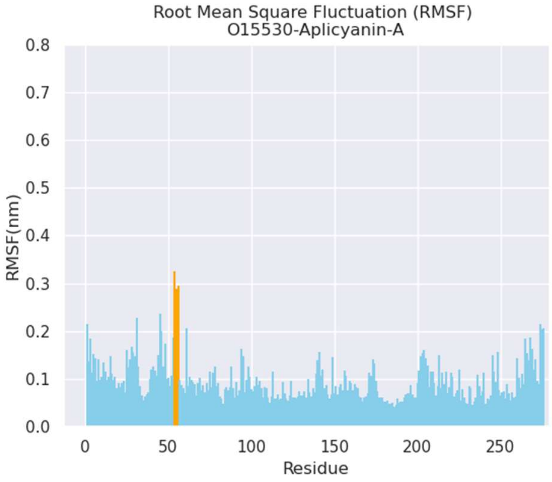

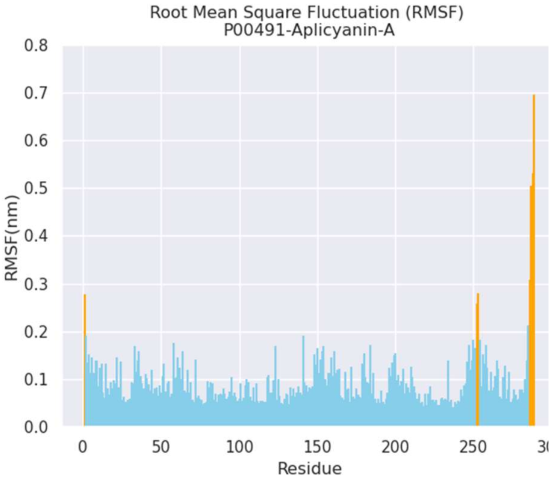

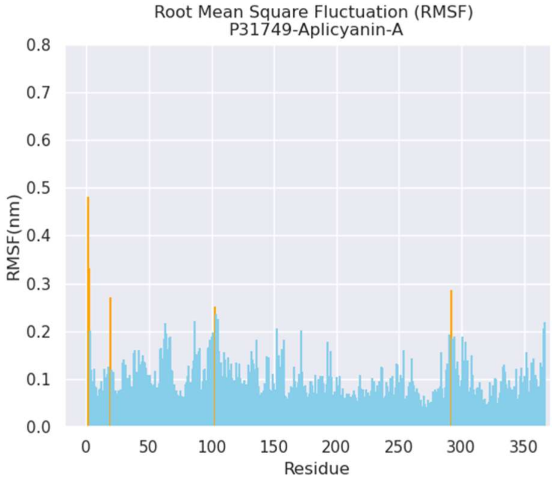
Root Mean Square Fluctuation (RMSF)
P31749-Aplicyanin-A
RMSF(nm)
Residue

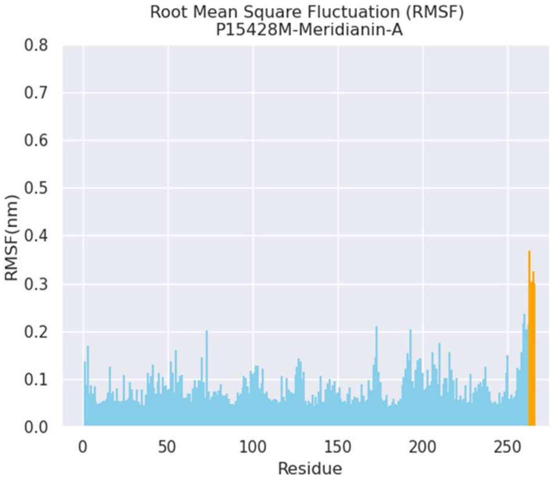
Root Mean Square Fluctuation (RMSF)
P15428M-Meridianin-A
RMSF(nm)
Residue

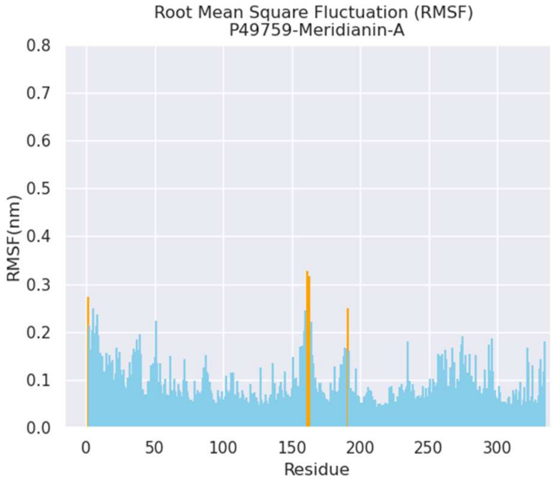
Root Mean Square Fluctuation (RMSF)
P49759-Meridianin-A
RMSF(nm)
Residue

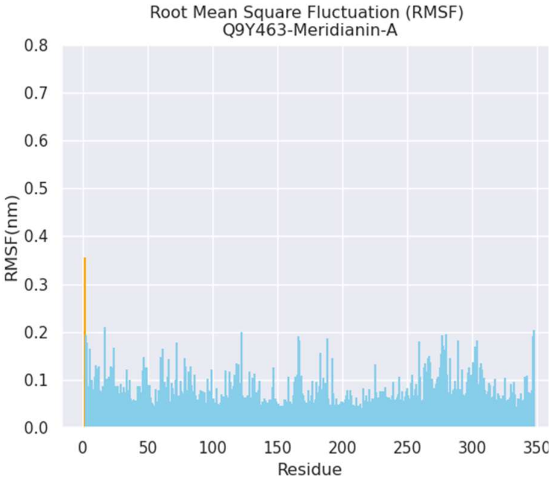
Root Mean Square Fluctuation (RMSF)
Q9Y463-Meridianin-A
RMSF(nm)
Residue

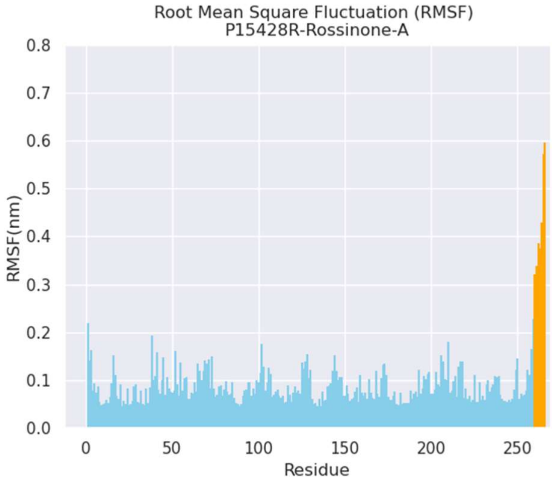
Root Mean Square Fluctuation (RMSF)
P15428R-Rossinone-A
RMSF(nm)
Residue

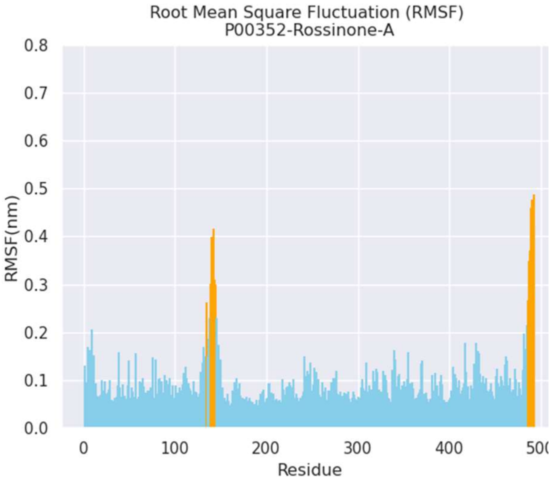
Root Mean Square Fluctuation (RMSF)
P00352-Rossinone-A
RMSF(nm)
Residue

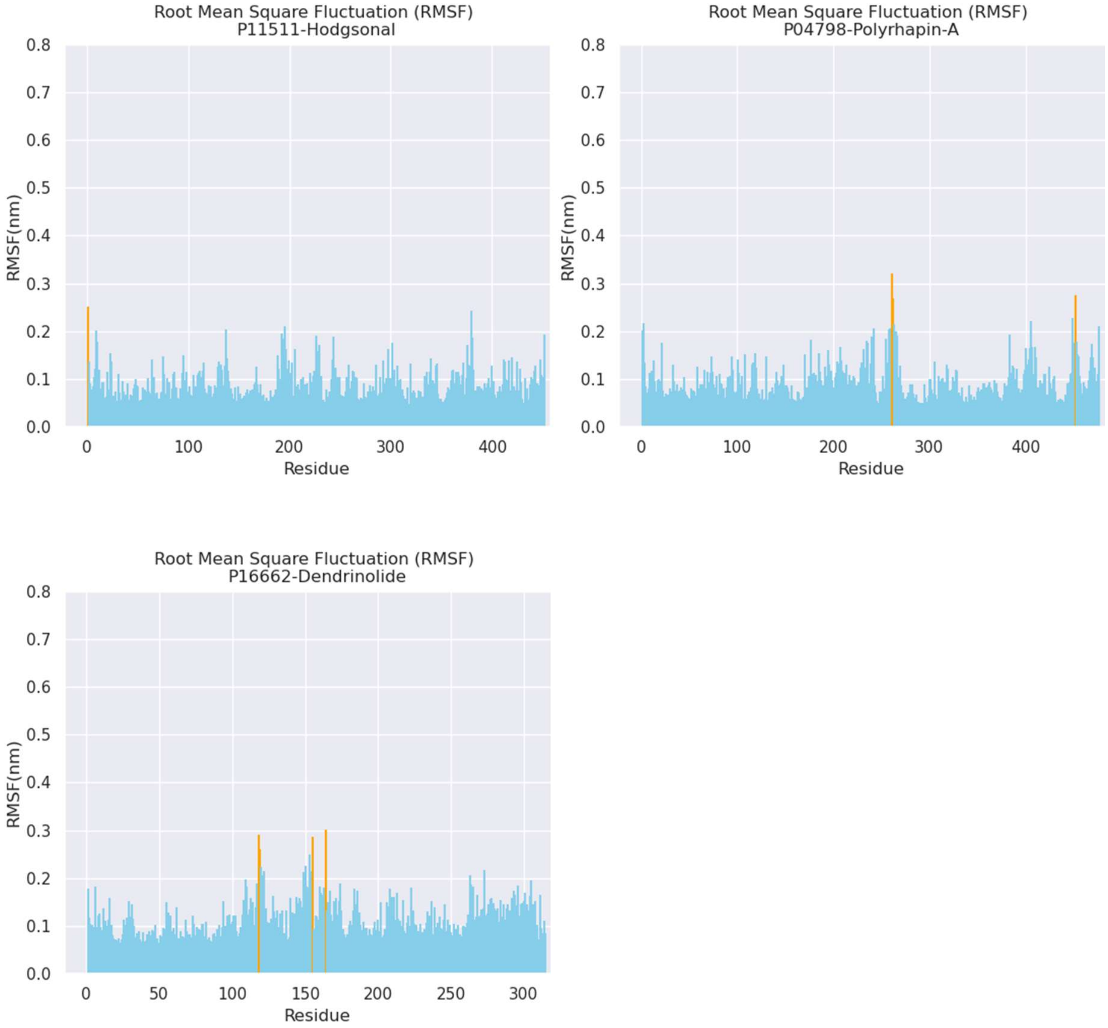

Figure S7. Root-mean-square fluctuation (RMSF) per residue (X-axis) values of each system along the MD simulation. The highest fluctuations (> 0.25 nm) detected have been highlighted in orange. Residue number corresponds to its position (Nterm-Cterm) in the simulated target sequence.
